# Changes in the Microbial Composition of the Rhizosphere of Hop Plants Affected by Verticillium Wilt Caused by *Verticillium nonalfalfae*

**DOI:** 10.3390/microorganisms11071819

**Published:** 2023-07-16

**Authors:** Elena Gallego-Clemente, Víctor Moreno-González, Ana Ibáñez, Carla Calvo-Peña, Seyedehtannaz Ghoreshizadeh, Sebastjan Radišek, Rebeca Cobos, Juan José R. Coque

**Affiliations:** 1 Instituto de Investigación de la Viña y el Vino, Escuela de Ingeniería Agraria, Universidad de León, 24009 León, Spain; mgallc00@estudiantes.unileon.es (E.G.-C.); aibas@unileon.es (A.I.); ccalp@unileon.es (C.C.-P.); sghore00@estudiantes.unileon.es (S.G.); 2 BioDatev, 24195 Villaobispo de las Regueras, Spain; vmorg@unileon.es; 3 Departamento de Biodiversidad y Gestión Ambiental, Universidad de León, 24071 León, Spain; 4 Slovenian Institute of Hop Research and Brewing, 3310 Žalec, Slovenia; sebastjan.radisek@ihps.si

**Keywords:** hop, Verticillium wilt, soil, rhizosphere, bacterial populations, fungal populations, metataxonomy

## Abstract

Verticillium wilt is a devastating disease affecting many crops, including hops. This study aims to describe fungal and bacterial populations associated with bulk and rhizosphere soils in a hop field cultivated in Slovenia with the Celeia variety, which is highly susceptible to *Verticillium nonalfalfae*. As both healthy and diseased plants coexist in the same field, we focused this study on the detection of putative differences in the microbial communities associated with the two types of plants. Bacterial communities were characterized by sequencing the V4 region of the 16S rRNA gene, whereas sequencing of the ITS2 region was performed for fungal communities. The bacterial community was dominated by phyla Proteobacteria, Acidobacteriota, Bacteroidota, Actinobacteriota, Planctomycetota, Chloroflexi, Gemmatimonadota, and Verrucomicrobiota, which are typically found in crop soils throughout the world. At a fungal level, *Fusarium* sp. was the dominant taxon in both bulk and rhizosphere soils. *Verticillium* sp. levels were very low in all samples analyzed and could only be detected by qPCR in the rhizosphere of diseased plants. The rhizosphere of diseased plants underwent important changes with respect to the rhizosphere of healthy plants where significant increases in potentially beneficial fungi such as the basidiomycetes *Ceratobasidium* sp. and *Mycena* sp., the zygomycete *Mortierella* sp., and a member of Glomeralles were observed. However, the rhizosphere of diseased plants experienced a decrease in pathogenic basidiomycetes that can affect the root system, such as *Thanatephorus cucumeris* (the teleomorph of *Rhizoctonia solani*) and *Calyptella* sp.

## 1. Introduction

Verticillium wilt is a vascular disease affecting many different economically important crops all around the world, such as cotton, potato, sunflower, woody perennials [1], hop [2], and olive [3]. The primary pathogenic species which cause Verticillium wilt are *V. dahliae*, *V. albo-atrum* (sensu lato), and *V. longisporum* [4]. They are soil-borne fungal pathogens able to survive in soils for long periods of time due to the production of resting structures such us microsclerotia, chlamydospores, or resting mycelia [1]. They infect plants through their roots, reaching the vascular system (xylem), which they use to spread the infection throughout the plant [5].

Hop (*Humulus lupulus* L) is susceptible to Verticillium wilt caused by *V. dahliae* and *V. nonalfalfae* (formerly *V. albo-atrum*). Most strains of *V. dahliae* and *V. nonalfalfae* which infect hop plants cause typical symptoms of Verticillium wilt, like yellowing, curling, wilting, necrosis of lower leaves, and swelling of bines which, when cut open, exhibit a prominent medium- to dark-brown discoloration of the vascular tissues [6]. More worrisome is the infection by lethal strains (pathotypes) of *V. nonalfalfae* causing a complete dieback of rootstock [7].

Currently, no effective treatments for Verticillium wilt of hop are available for farmers. Several factors contribute to this fact, including that once the pathogen reaches the vascular system, the chances of curing the disease are almost nil. Therefore, perhaps the best way to combat this pest is to prevent the plants from becoming infected in the first place. Given the soil-borne nature of these pathogenic fungi and the fact that the infection takes place at the root-system level, correct soil management could contribute to mitigating this disease.

Soil is a very complex microbial ecosystem where huge microbial populations develop and where very complex interactions take place [8]. Understanding soil microbiota can be particularly interesting in order to identify other microbiological factors that make a plant more susceptible to disease, as well as possible antagonistic microorganisms that can act as biocontrol agents (BCA), or even to investigate the possible factors that lead to the existence of suppressive soils [9,10]. Studies on the interaction between soil microbiota and plants have attracted worldwide interest and attention because of the need for restoration and maintenance of biodiversity, which are priority issues in every conservation policy [10]. In this context, metagenomic analyses of soils, defined as the study and exploration of the collective genomes present in a particular soil sample, and metataxonomic analyses (a high-throughput process using markers highly conserved across taxa, like 16S rRNA for bacteria and ITS region for fungi) have become important tools for unraveling and characterizing the high complexity of the microbiota associated with a particular microbial ecosystem [11] and a source for the identification of putative BCAs, as reviewed by Cobos et al. [12].

Roots are surrounded by a narrow zone (often only a few millimeters thick) of soil known as the rhizosphere, which contains microbial populations different from those found in the surrounding bulk soil. This is because the plant roots release a huge variety of chemical compounds known as rhizodeposits (nutrients, border cells, exudates, and mucilage) which make the rhizosphere a much more nutritive ecological niche than the bulk soil. Other factors contributing to intense microbial activity in the rhizosphere are its more stable ambient conditions and higher water availability. In fact, plants shape their rhizosphere microbiome, as evidenced by the fact that different plant species host different microbial communities when grown in the same soil [13,14]. Even the same plant species can harbor different microbial communities in the rhizosphere depending on the rootstock used [15,16].

Biocontrol of soil-borne pathogens has long been a goal of sustainable agriculture [17]; however, due to several factors, such as the high complexity and variability of the resident microbial communities, few commercial biocontrol products against soil-borne diseases in agriculture are available [18]. Thus, to ensure that introduced BCAs can thrive in the soil environment and efficiently fight fungal pathogens over time, a deep knowledge of how the introduced BCAs interact with soil-borne pathogens, and other soil microorganisms, is required. This knowledge may assist in improving the survival of the introduced BCAs and in increasing the suppressiveness of soil microbiomes against soil-borne fungal pathogens.

This study focuses on characterization of the bacterial and fungal microbiota associated with bulk and rhizosphere soils sampled from both healthy and diseased plants in a hop field showing Verticillium wilt caused by *V. nonalfalfae*. Based on Illumina sequencing of the fungal internal transcribed spacer (ITS) region and the bacterial 16S rRNA gene, changes in bacterial and fungal soil communities were investigated in both types of soils. Special attention was paid to analyzing significant changes in microbial populations, particularly fungi, in the rhizosphere of diseased plants in relation to healthy plants, and their possible influence on the development of the disease. To our knowledge, this is the first study showing new insights from analyzing fungal and bacterial communities associated with Verticillium wilt of hop in diseased and healthy soils.

## 2. Materials and Methods

### 2.1. Field Description and Sampling

In the second week of June 2022, soil and plant samples were collected from a highly susceptible variety of Celeia (10-year-old plants) in an infected hop field seeded with Verticillium wilt (*V. nonalfalfae).* The hop field was located in the Savinja valley, Slovenia (GPS: Y 50°61′65.424/X 12°39′75.556) and cultivated according to conventional agronomic practice. Hop plants exhibiting symptoms of Verticillium wilt and non-symptomatic plants were randomly selected in the effected part of the hop field (Supplementary material; Figure S1).

Samples were collected from 10 symptomatic and 10 non-symptomatic plants which were in phenological stage BBCH 38. Bulk soil samples were taken 20–30 cm away from the base of the plant. The 5 cm superficial soil was removed, and bulk soil samples were taken at a depth of 10–15 cm. Sampling of rhizosphere soil was taken after digging to access the root system of the plants. After that, the exposed rhizosphere soil in direct physical contact with the roots was collected. Bulk and rhizosphere soil samples were transferred to sterile Falcon tubes (50 mL), kept in an icebox, and preserved at 4 °C until processing for genomic DNA extraction. Soil samples for performing standard physicochemical characteristics were taken as described for bulk samples (approximate volume 200 g/plant) and pooled into representative samples for symptomatic and non-symptomatic plants. Pooled soil samples were frozen and stored at −20 °C for physiochemical analysis.

To detect *V. nonalfalfae,* plant samples were collected from each of the 20 selected plants. The samples were from the basal section of hop bines (20 cm in length) which grew from the root system adjacent to the rhizosphere sampling. The plant samples were collected in plastic bags and immediately transferred to the laboratory for mycological analysis.

### 2.2. Verticillium Detection in Plants

Plant samples were analyzed according to EPPO diagnostics protocol PM7/78 (2) for detection of V. nonalfalfae and V. dahliae [19]. The hop bines were surface sterilized (30 s, 70% ethanol), and then the epidermis was peeled away using a sterile scalpel. Four pieces (1 cm) of xylem tissue per bine were cut off and placed on potato dextrose agar (PDA) amended with 500 ppm of streptomycin sulphate. After 14 days at 22 °C in the dark, growing colonies were subjected to culture identification. Verticillium isolates were identified based on morphological characteristics described by Inderbitzin et al. [20].

### 2.3. Soil Physicochemical Properties

Physicochemical properties of pooled bulk soil were analyzed in the Department for Agrochemistry and Brewing at the Slovenian Institute of Hop Research and Brewing (Žalec, Slovenia). Briefly, determination of soil texture and mechanical analysis were conducted according to the standard sedimentation method, with particle size distribution in a combination of sieving and sedimentation techniques (ISO 11277:2020 [21]). Soil pH was analyzed in 1M potassium chloride solution (ISO 11464:2006 [22]); P_2_O_5_ and K_2_O were analyzed in the ammonium acetate lactate extract according to Egnér et al. [23] using UV/VIS spectrophotometry for phosphorus, and atomic absorption spectrophotometry for potassium determination. Copper (Cu), zinc (Zn), iron (Fe), and Manganese (Mn) were determined using atomic absorption spectrometry (AAS) in an air-acetylene flame [24]. Magnesium was analyzed with the CaCl_2_ method [25] and determined by AAS. Available boron was measured according to the standard NF X31-122, and humus content according to the ISO 14235:1999 [26]. Total nitrogen (N) was analyzed by the modified Kjeldahl method (ISO 11261:1996 [27]), and nitrate (NO_3_^−^) content was determined using high-performance liquid chromatography (HPLC) using the standard ISO 14255:1998 [28].

### 2.4. Quantification of Verticillium *sp.* in Soil Samples by qPCR

DNA from soil samples was isolated by using the DNeasy PowerSoil kit (Qiagen, Hilden, Germany) following the manufacturer’s instructions and quantified using a Qubit 2.0 Fluorometer (Thermo Fischer Scientific, Pleasanton, CA, USA). The presence of *V. dahliae* and *V. nonalfalfae* was quantified by qPCR performed in the Mx3005P qPCR System (Stratagene, San Diego, CA, USA) using the SYBR TB Green Premix Ex Taq (Takara). Specific primers for qPCR detection of *V. dahliae* (Vdf 5′-GGCTCAAGTTAACTACGG-3′ and Vdr 5′-CTGTCATGTATATAAGATACTACTG-3′) and *V. nonalfalfae* (Vnaf 5′-GGCTTTTGCTTTCTCTTG-3′ and Vnar 5′-GACCAAATGTAATTGTCCAG-3′) were used [29]. The qPCR reactions were carried out in triplicate in 96-well plates in a 20 μL final volume containing 1× TB Green Premix Ex Taq (Takara, Saint-Germain-en-Laye, France), 200 nM of each primer, and 5 μL of soil sample DNA. The thermal cycling conditions were initial denaturation at 95 °C for 2 min, followed by 40 cycles of denaturation at 95 °C for 20 s and annealing and elongation at 63 °C for 20 s. Threshold cycle (Ct) values were calculated using MxPro-Mx3005P v4.10 Stratagene software. The standard curves for qPCR were constructed using 10-fold serial dilutions of *V. dahliae* V937I and *V. nonalfalfae* (VNAT2) DNA as template. Three independent experiments were carried out with three reactions of each concentration to generate the standard curves. Standard curves were generated by plotting Ct values of the qPCR assays against the corresponding logarithm (base 10) of the concentrations. Distilled water was used as a template for the negative control.

### 2.5. DNA Extraction and Sequencing

DNA from soil samples was isolated as indicated above. The ITS2 region located inside the fungal nuclear ribosomal DNA (rDNA) was amplified using the primers ITS3_KYO2 and ITS4 [30], and the bacterial V4 region of the *16S rRNA* gene was amplified using the 515F and 806R primers [31].

DNA amplicon libraries were generated using the next limited cycle PCR: initial denaturation at 95 °C for 32 min, followed by 28 cycles of annealing (95 °C 30 s, 58 °C 30 s, 72 °C 30 s) extension at 72 °C 5 min, using a KAPA HiFi HotStart ReadyMix (Roche, Indianapolis, IN, USA). Then, Illumina sequencing adaptors and dual-index barcodes (Nextera XT index kit v2, FC-131-2001) were added to the amplicon. Finally, libraries were normalized and pooled prior to sequencing.

The pool containing indexed amplicons was loaded on the MiSeq reagent cartridge v3 (MS-102-3003) spiked with 25% PhiX control to improve base calling during sequencing, as recommended by Illumina for amplicon sequencing. Sequencing was conducted using a paired-end, 2300 pb cycle run on an Illumina MiSeq sequencing system.

### 2.6. Data Analysis of the High-Throughput Amplification Assay

The forward and paired reads were filtered and trimmed with TRIMOMMATIC v.0.39 [32] (parameters: CROP:280 TRAILING:28 SLIDINGWINDOW:10:28 MINLEN:100) based on the quality analyses carried out with FastQC v.0.11.9 [33].

With the preprocessed and filtered files, we proceeded to identify the sequences using the mothur v.1.48.04 pipeline [34]. Sequences whose length was not between 300 and 450 base pairs for ITS reads, or between 230 and 300 base pairs for 16S reads, were removed. Subsequently, the unique sequences were extracted and chimeric sequences were identified and filtered using UCHIME [35]. Finally, identification was based on the UNITE v.65 database for ITS reads [36] and SILVA nr v.138-16 for 16S reads [37] by using mothur software [34]. After identification, the sequences were grouped into OTUs with 95% similarity, on which a taxonomic assignment was made.

Non-fungal and non-bacterial sequences were removed for subsequent analyses. Finally, for the OTUs marked as ‘unidentified’ at the family or genus level among the 200 most abundant in each dataset (Fungi and Bacteria), a complementary BLASTN identification was performed. This identification was carried out against the targeted loci (Fungi ITS and Bacteria 16S, respectively) NCBI database in order to refine genus-level identifications. Only BLASTN identifications with more than 70% of the first 100 hits belonging to one single genus and a mean identity above 0.85 were considered.

### 2.7. Microbial Diversity, Taxonomy Distribution, and Statistical Analysis

α-diversity was estimated with Chao1 and Shannon indices by means of the Phyloseq package [38]. In addition, the Pielou’s index [39] was computed by dividing the Shannon index by the logarithm of observed richness. These indices were fitted to a generalized linear model (GLM) with a Gamma error distribution with a logarithmic link function suing the MASS package v.7.3-58 [40]. In all models, the effects of the soil type (bulk/rhizosphere) and their condition (healthy or diseased) were analyzed, as was the interaction between both factors. When no significant interaction was found, it was removed. Finally, for significant factors, a post hoc pairwise comparison of the estimated marginal means was performed with emmeans v.1.7.5 [41], adjusting the *p*-value with the FDR method [42].

To analyze β-diversity, firstly, a PERMANOVA [43] with 9999 permutations was carried out to check if the composition of the communities depends on the experimental situation. Subsequently, a PCoA was carried out using the Bray–Curtis distance. Based on these results, the differentiation of the samples according to the study condition in the first three dimensions was verified using ANOVA tests. All these analyses were carried out with phyloseq v.1.40.07 and vegan v.2.6-28 packages [38,44].

Finally, in order to verify the effects of the soil type and its condition on the bacterial and fungal populations, a pairwise differential analysis for count data was carried out with the DESeq2 v.1.36.0 package [45]. The *p*-values were adjusted with the FDR method [42].

All analyses were conducted in R v.4.2.1 [46] with RStudio 2022.07.2 Build 576 in an Ubuntu 20.04.5 LTS system with 64 GiB RAM and 16 cores.

## 3. Results

### 3.1. Verticillium Detection in Plants and Soil Physiochemical Analysis

Xylem excision from hop bines revealed typical xylem browning associated with Verticillium wilt in all 10 samples collected from symptomatic plants, while non-symptomatic plants exhibited normal white xylem tissue. Xylem mycological analysis confirmed the presence of V. nonalfalfae in symptomatic plants, whereas no presence of V. nonalfalfae was found in non-symptomatic plants. No other fungal cultures were found in any of the sampled plants.

Physiochemical analysis of pooled bulk soil samples taken in the proximity of diseased and healthy plants revealed no differences in soil texture and mechanical characteristics. Both soil samples were categorized as medium-heavy soil. However, significant differences were found at the chemical level, as the sample from diseased plants revealed significantly higher levels of total nitrogen (160%), nitrate (NO_3_^−^) (66%), potassium (44%), and trace elements such as magnesium (57%), manganese (53%), and iron (13%) (Table 1). High soil nitrogen levels near diseased plants is a consequence of disturbed nitrogen uptake since diseased plants do not continue growing but rather wilt and die. As can be seen in the Supplementary Materials (Figure S1), growth of diseased plants is severely affected, compared to healthy plants, so their ability to remove nutrients from the soil decreases significantly. Meanwhile, healthy plants in phenological stage BBCH 38 show rapid vegetative growth and high nitrogen uptake. Higher soil nitrogen content can consequently result in increased levels of some trace elements [47], as seen in our study in the case of diseased plants.

### 3.2. Overview of Bacterial and Fungal Populations

After paired-end alignments; quality filtering; and deletion of chimeric, singletons, mitochondrial, and chloroplast sequences, a total of 1,680,920 sequences belonging to 3187 OTUs were identified in fungi (Supplementary Materials Table S1) and 3,182,993 sequences belonging to 34,622 OTUs were identified in bacteria (Supplementary Materials Table S2).

**Figure 1:**
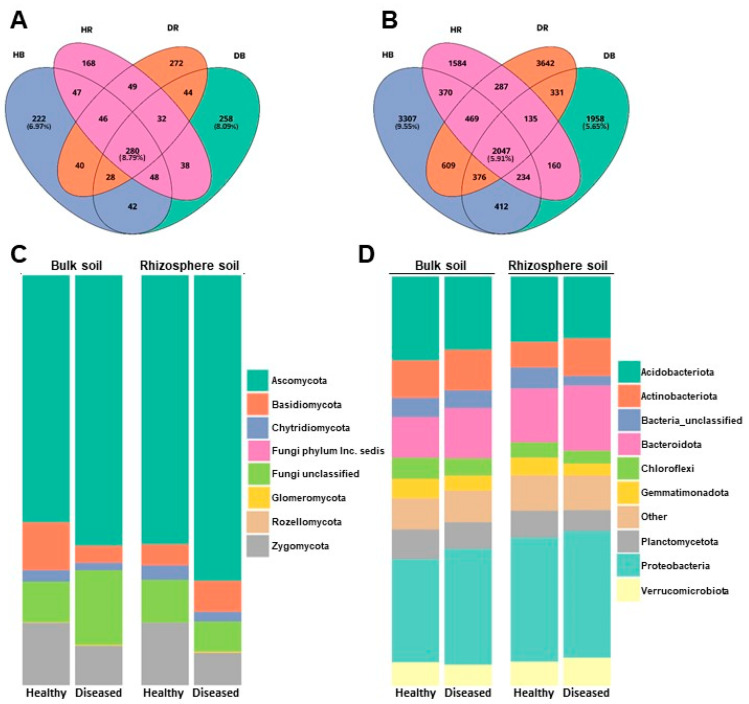
Venn diagram illustrating the overlap in number of OTUs identified in fungal (**A**) and bacterial (**B**) communities in samples, and representation of the relative abundances of the most represented fungal (**C**) and bacterial (**D**) phyla in the two soil types (bulk and rhizosphere) and the two conditions (healthy and diseased) analyzed.

The core of the fungal community is made up of 280 OTUs, which represent 8.79% of the total fungal OTUs, while the core of the bacterial community is made up of 2047 OTUs, which correspond to 5.91% of the bacterial OTUs detected. The percentage of unique OTUs for each health condition (healthy or diseased) ranged between 6.97% and 8.09% in fungi and between 9.55% and 5.65% in bacteria (Figure 1A,B).

The relative concentration of the most abundant bacterial and fungal phyla detected across all samples is shown in Figure 1C,D. The fungal community is composed of 8 phyla, the most abundant on average being Ascomycota, accounting for 66.05 ± 8.43% of all sequences, followed by Zygomycota (14.36 ± 6.42%), Basidiomycota (6.57 ± 3.02%), and Chytridiomycota (2.38 ± 0.81%). Approximately 10.39 ± 5.57% of fungal sequences/OTUs were not identified. Other fungi, belonging to Phyla Glomeromycota (0.22 ± 0.14%), Rozellomycota (0.02 ± 0.01%), and Incertae sedis Phyla (0.0001 ± 0.0005%), were represented to a lesser extent (Figure 1C).

**Figure 2:**
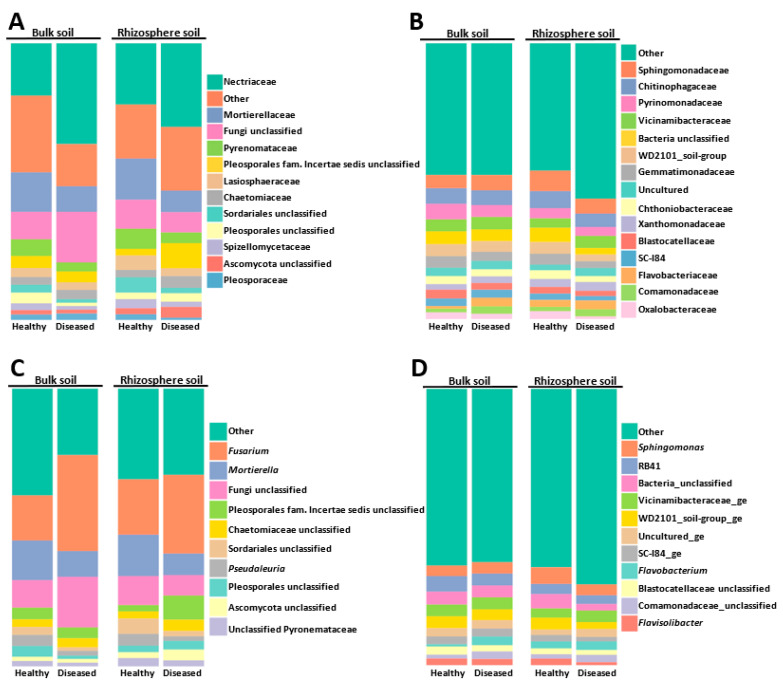
Abundance of fungal (**A**,**C**) and bacterial (**B**,**D**) communities at the level of family (**A**,**B**) and genus (**C**,**D**). “Other” includes all other families or genera, with a relative abundance of less than 2% in the global population.

The most abundant families and genera represented in the global microbial community are shown in Figure 2. The fungal community was widely dominated by members of the Ascomycota families, of which the Nectriaceae and Pyronemataceae taxa were the most abundant, with only the Mortierellaceae (Zygomicota) family sneaking into these first positions. Nectriaceae members increased in bulk and rhizosphere diseased soils, whereas the opposite behavior was detected for Mortierellacea, whose relative abundance decreased in diseased soils (Figure 2A).

When analyzing the global fungal community, we could verify that the 100 most abundant OTUs represented 88.80% of the total detected OTUs, whereas coverage of the 200 most abundant OTUs increased to 94.38%.

At the genera level, the ascomycete *Fusarium* fungal community was dominant (Figure 2C) (20 OTUs that represented at least 22.53% of the total fungal community), with relative abundances ranging between 15.87% in diseased rhizosphere (DR), 22.07% in healthy bulk soil (HB), 23.21% in diseased bulk soil (DB), and 23.77% in healthy rhizosphere (HR). It should be noted that by using mothur software, Otu0001 was identified as “Nectriaceae unclassified” (Supplementary Materials Table S1). However, more detailed analyses using BLASTN allowed us to confirm that Otu0001 corresponded to the *Fusarium* genus. Since the BLASTN analysis was performed only for the 200 most abundant OTUs, we cannot rule out that the relative abundance of *Fusarium* was slightly higher in the total population analyzed, as some OTUs initially annotated as “Nectriaceae unclassified” by the mothur software might belong to this genus. The second most abundant genus was the zygomycete *Mortierella* (represented by 179 OTUs that corresponded to 14.11% of the total fungal community) (Figure 2C). Members of the Pyronemataceae family of Ascomycota were also abundant and were represented by *Pseudaleuria* sp. (Otu0010) and an unclassified Pyronemataceae (Otu008) (Figure 2C and Supplementary Materials Table S1). Together, the *Fusarium* sp., *Mortierella* sp., and *Pseudaleuria* sp. genera represented 39.57% of the global fungal community.

*Verticillium* was detected at a very low rate, representing only 0.009% of the OTUs detected in the total analyzed soil samples. The presence of *Verticillium* in the samples tested by soil type (bulk or rhizosphere soil) and condition (healthy or diseased soil) was also low, with levels ranging from 0.0014% in DB soil, 0.0024% in HB soil, 0.0041% in HR, and increasing up to 0.0268% in DR soil. On average, its presence in DR soil was between 6.4 and 18.9 times higher than those detected in the other samples analyzed. There is some correspondence between these data and the detection of *V. dahliae* by quantitative PCR (qPCR), which was negative for all soil types and conditions tested (HB, DB, HR, and DR). In contrast, by performing a similar analysis, *V. nonalfalfae* was not detected in HB, DB, or HR samples, but this species could be detected in 60% of the DR samples analyzed, at levels ranging from 0 to 16 pg DNA/g soil (average of 6.40 ± 6.69 pg DNA/g soil).

The bacterial community was composed of 43 phyla, with Proteobacteria accounting for 26.98 ± 3.34% of all sequences, followed by Acidobacteriota (17.30 ± 3.75%), Bacteroidota (13.18 ± 4.82%), and Actinobacteriota (9.92 ± 1.77%). These four phyla represented more than 67% of the total number of bacterial taxa detected. Phyla Planctomycetota (6.41 ± 0.96%), Verrucomicrobiota (5.76 ± 0.90%), Chloroflexi (4.34 ± 0.82%), and Gemmatimonadata (3.95 ± 0.64%) also appeared in appreciable numbers, although at levels lower than those of the four dominant phyla (Figure 1D). Approximately 4.01 ± 0.77% of sequences/OTUs were not identified (Supplementary Materials Table S2). Phylum Firmicutes only represented 1.03 ± 0.59% of the total OTUs detected.

When analyzing the bacterial community, we observed that the 100 most abundant OTUs represented 61.86% of the total OTUs detected, whereas coverage of the 200 most abundant OTUs increased up to 74.28%. The most abundant bacterial families were Sphingomonadaceae (Alphaproteobacteria), Chitinophagaceae (phylum Bacteroidota), and the Pyrinomonadaceae and Vicinamibacteraceae families of the Acidobacteriota phylum (Figure 2B).

At a genera level, the *Sphingomonas* genus, a member of the Alphaproteobacteria, was the dominant taxon, representing 4.71% of the total OTUs detected. Members of the Acidobacteria *RB41* clade were also abundant. Other abundant bacteria, at the genus level, were the members of the phylum Bacteroidota *Flavobacterium* (3.30%) and *Flavisolibacter* (1.89%) (Figure 2D). Other interesting genera, although detected at a rate lower than 2% in the global bacteria community, were the members of Gammaproteobacteria class *Pseudomonas* (1.69%) and *Massilia* (1.28%). Regarding Actinobacteriota, the most represented genera were *Pseudarthrobacter* (1.58%), *Gaiella* (0.70%), *Nocardioides* (at least 0.72% since Otu00061 was identified by BLAST, Supplementary Materials Table S2), and *Streptomyces* (at least 0.70% since Otu00152 was identified by BLAST; Supplementary Materials Table S2).

### 3.3. Analysis of the α-Diversity of Microbial Populations

**Figure 3:**
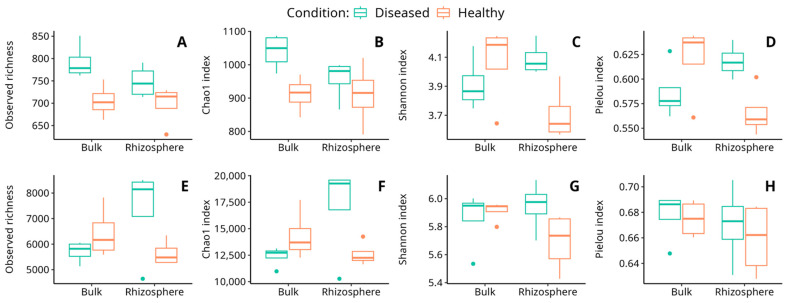
Variation of observed richness (**A**,**E**), Chao1 index (**B**,**F**), Shannon index (**C**,**G**), and Pielou index (**D**,**H**) for the fungal (**A**–**D**) and bacterial (**E**–**H**) communities in the soil samples analyzed. Dots observed in some graphs correspond to outlier values.

In the fungal community, the results showed that the observed richness and Chao1 index were significantly affected by the soil condition, with a significantly higher richness in diseased soils than in healthy ones. On the other hand, the Shannon and Pielou’s Evenness indices showed significant interaction of the soil type and its condition (Table 2; Figure 3). According to these results, the diversity in rhizosphere soils increased in the diseased condition with respect to the healthy one (Supplementary Materials Table S3). Thus, despite the fact that the plant’s health condition is significantly related to fungal richness, soil fungal diversity was observed to be significantly related to the interaction of both soil type and condition.

In bacteria, the results showed that the observed richness and the Chao1 index, as well as the Shannon index, present at least a significant influence on some factors (Table 2, Figure 3). In all three cases, a significant interaction between the soil type and its condition was detected, indicating that the influence of these two factors on soil richness is not independent (Supplementary Materials Table S4).

### 3.4. Analysis of the β-Diversity of Microbial Communities

In the fungal community, the PERMANOVA test (permutational multivariate analysis of variance) shows that community composition was significantly affected by the soil type and its condition (Table 3), although a significant interaction was not found.

**Figure 4:**
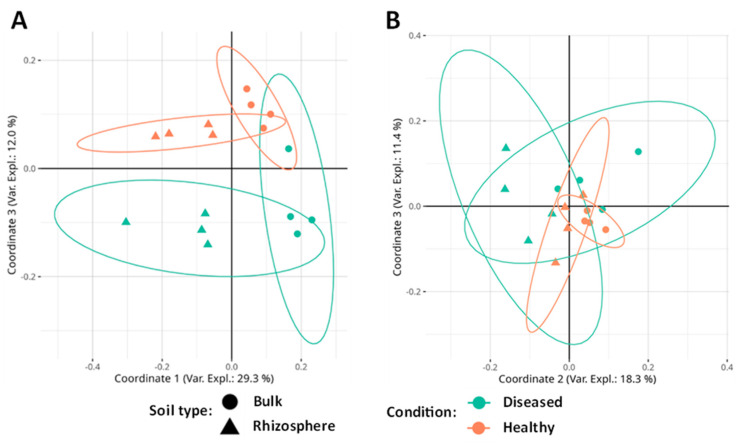
(**A**) PCoA analysis based on Bray–Curtis distance applied to fungal community data. (**B**) PCoA analysis based on Bray–Curtis distance applied to the data of the bacterial community. Ellipses indicate a 95% confident interval.

The principal coordinates analysis (PCoA) significantly separates the soil types (bulk or rhizosphere) in the first coordinate (29.3% of the variance explained) and the conditions (diseased/healthy) in the third coordinate (12.0% of the variance). Coordinate 2 (16.8% of the variance) shows a significant interaction between the soil type and its diseased or healthy condition. These results suggest that the greatest differences in terms of fungal community composition are due to soil type, although there are also common ecological patterns in terms of soil condition (Figure 4A).

In the case of bacteria, the PERMANOVA test shows that community composition is significantly affected by the soil type and the presence or absence of disease (Table 3). No significant interaction was found between the factors. The PCoA shows that diversity is influenced by soil type in coordinate 2 (18.3% of the variance) and by its condition in coordinates 2 (significant interaction) and 3 (11.4% of the variance). Coordinate 1 (33.9% of the variance) does not separate by any factor (Figure 4B).

### 3.5. Differential Abundances

In the fungal community, 21 OTUs were found with significantly different abundances between healthy bulk and rhizosphere soils, of which only four presented an LFC > 0, which indicates that their frequency increases in bulk soil with respect to rhizosphere soil. Among them, the basidiomycete *Entoloma* (Otu0032) stands out. In the OTUs with an LFC < 0, and therefore whose frequency decreases in bulk soil compared to rhizosphere, the Agaricomycetes *Calyptella* (Otu0163; LFC = −23.56) and *T. cucumeris* (Otu0116; LFC = 23.97) stand out (Supplementary Materials Table S5). Although initially and according to a mothur search, Otu0116 had been identified as *Thanatephorus* unclassified, a more careful analysis using a BLASTN search allowed us to identify Otu0116 as *T. cucumeris* (in fact, 94 out of the first 100 hits were reported as *T. cucumeris* or *R. solani*). *T. cucumeris* is the teleomorph of the important fungal phytopathogen *Rhizoctonia solani* [48]. 

When a comparison between diseased bulk soil and diseased rhizosphere soil was made, 12 OTUs exhibited significantly different abundances. Four OTUs showed an LFC > 0, including members of the chytridiomycete *Powellomyces* (Otu0159; LFC = 22.51), the zygomycete *Mortierella* (Otu0228; LFC = 21.45), and the ascomycete *Lasiosphaeris* (Otu0211; LFC = 20.84). Among the OTUs with an LFC < 0, we can highlight the ascomycete *Xylaria* (Otu0358; LFC = –21.52) (Supplementary Materials Table S6).

When comparing healthy and diseased bulk soils, a total of 40 OTUs exhibited significant different abundances, of which only 6 have an LFC > 0 (Supplementary Materials Table S7). Among the OTUs with a negative LFC value, whose proportion in healthy versus diseased bulk soils is therefore diminished, we can mention the presence of several OTUs of the basidiomycetes *Mycena*, *Ceratobasidum* (Otu0056), or *Mortierella* (Otu0128) (Supplementary Materials Table S7).

Finally, regarding a comparison between healthy and diseased rhizosphere soils, 37 OTUs exhibited significantly different abundances. For a total of 15 OTUs, the LFC value was positive, and therefore, its abundance was significantly higher in the rhizosphere of healthy plants. The four taxa present in significantly more abundance in the rhizosphere of healthy plants compared to the rhizosphere of diseased plants were basidiomycetes of the class Agaricomycetes, including members of *Calyptella* (Otu0163) and *T. cucumeris* (Otu0116). However, among those taxa whose frequency is significantly higher in the rhizosphere of diseased individuals (LFC < 0), we also have several basidiomycetes of the class Agaricomycetes, including several members of the genus *Mycena* (Otu0453, Otu0493, and Otu0344) and *Ceratobasidium* sp. (Otu0056) and (Supplementary Materials Table S8).

In the bacterial community, 13 OTUs with significantly different abundances between HB and HR soils were found, 48 OTUs between DB and DR, 8 OTUs between HB and DB soils, and 29 OTUs between HR and DR soils (Supplementary Materials Tables S9–S12, respectively). In this last case, we observed a significant increase in the relative abundances of several OTUs belonging to the families Gemmatimonadaceae (Otu0476) and Sphingobacteriaceae (Otu01230), the most relevant (Supplementary Materials Table S12) in the rhizosphere of diseased plants.

## 4. Discussion

Hop is a very important crop widely cultivated in the US Northwest and several European countries. Its fundamental purpose lies in the use of its female flowers, or cones, to produce beer, to which they provide bitterness and other aromas. As a member of the Cannabiaceae family, it is also an important source of natural products with antioxidative, antimicrobial, and antigenotoxic potentials, and therefore has a wide application in sectors such as medicine or cosmetics [49]. As with any crop, its profitability is threatened by different fungal pathologies, among which Verticillium wilt is one of the most important. *V. nonalfalfae* outbreaks are the cause of severe economic losses in hop fields in Slovenia [7], England [50], Germany [51], Belgium [52], and recently, in Czech Republic [53].

Understanding the different factors that can contribute to the development of any plant disease is vital for sustainable disease management. Among these factors, components that shape the rhizosphere community could be significant. In this context, we decided to analyze the fungal and bacterial population compositions in bulk and rhizosphere soils associated with both healthy and diseased plants. To our knowledge, this study is the first to investigate the microbial community composition of soils from a plot where healthy and diseased plants coexist.

### 4.1. Detection of Verticillium *sp.* in Bulk and Rhizosphere Soils

While, in general, we might think that Verticillium wilt severity could enhance significantly with increasing pathogen inoculum density [54], our data indicated that pathogen levels were extremely low in both bulk soil and rhizosphere soil. In fact, specific detection of *V. nonalfalfae* by qPCR was negative in both bulk soils (healthy and diseased) and in the rhizosphere of healthy plants. The pathogen could only be detected, and at very low levels, in 60% of the rhizosphere soils of diseased plants analyzed. Also, the metataxonomic analysis confirmed that *Verticillium* sp. was present at very low rates in all the soil samples analyzed, representing 0.0089% of the total fungal OTUs detected in the global population, and rates were lower in HB soil (0.0024%), DB soil (0.0014%), and HR soil (0.0041%). Higher levels (0.0264%) were detected in diseased rhizosphere soil samples, and on average, the DR levels were between 6.4 and 18.9 times higher than those detected in the other samples analyzed. Also, Wei and colleagues [54] reported that the level of *V. dahliae* inoculum was higher in the rhizosphere of diseased cotton plants than in the healthy plants, although such a difference explained only a small proportion of variation in wilt severities. These data suggest that an increase in the pathogen inoculum in the rhizosphere environment could allow effective plant infection, even though the relative abundance of the pathogen in soil is extremely low.

### 4.2. General Overview of Fungal Population

The global fungal community was widely dominated by members of the Ascomycota phylum, where the Nectriaceae and Pyronemataceae families were the most abundant, and only the Mortierellaceae family (Zygomycota) snuck into one of the first positions. Within the Nectriaceae family, *Fusarium* sp. was the most prevalent genus in both bulk and rhizosphere soils of any tested condition. *Fusarium* is a large genus of filamentous fungi, part of a group often referred to as hyphomycetes, which are widely distributed in soil and associated with plants, including several species which are harmful plant pathogens [55]. This fact is remarkable since the genus *Fusarium* includes numerous species of soil-borne fungal pathogens, which affect many crops, including hops. *F. avenaceum*, *F. sambucinum* [56], and *F. crookwellense* [57] are the main species affecting hop plants. Although these species were not detected in the *Fusarium* sp. subpopulation due to limitations of the technique used to discriminate at the species level, we cannot discard that they may be represented among the population of *Fusarium* sp. detected. *Mortierella* sp. was the second most widely represented fungal taxon. *Mortierella* sp. are considered saprotrophic microorganisms abundant in bulk soil, rhizosphere, and plant tissues. They are considered very valuable decomposers in agricultural soils due to key features such as their ability to survive under very unfavorable environmental conditions and to use carbon sources contained in polymers like cellulose, hemicellulose, and chitin. Also, some strains of this genus belong to the plant growth-promoting fungi (PGPF), and therefore, they are generally considered highly beneficial fungi [58]. The Pyronemataceae family was mainly represented by *Pseudaleuria* sp. This fungus has also been reported to be abundant in soils of oilseed rape crop, and its abundance and microbial activity increased in the rhizosphere environment [59], although unfortunately there are fewer data about its possible role in soils and the rhizosphere. Together, these three genera represented 39.57% of the global fungal community.

### 4.3. General Overview of Bacterial Population

When analyzing the global bacterial community, we noticed that the four most abundant bacterial groups found across all samples were Proteobacteria, Actinobacteria, Acidobacteria, and Bacteroidetes, which correspond to the top four reported by Lauber et al. [60] in natural ecosystems. The relative abundances of the seven dominant phyla found in our study (Acidobacteria, Actinobacteria, Bacteroidota, Chloroflexi, Gemmatimonadota, Planctomycetota, and Proteobacteria) correspond to those reported by Liu et al. [61] for agricultural soils in China planted with soybean, maize, or wheat. It has been suggested that bacterial community compositions, even at a coarse taxonomic level (i.e., phyla), are altered by agriculture [61], and that agricultural bacterial communities of perennial and annual systems are more similar to each other than to the bacterial communities found in natural ecosystems [62]. At the genus level, the most abundant taxon in the global community was *Sphingomonas* sp., a member of the Alphaproteobacteria that represented 4.71% of the total OTUs detected. Although this genus is known to be environmentally ubiquitous, studies have reported the pathogen antagonism and plant growth-promoting potentials of *Sphingomonas* [63]. Acidobacteria were also well represented by non-identified taxons of the Vicinamibacteraceae and Blastocatellaceae families which are abundant in a wide variety of soils. Members of Vicinamibacteraceae are characteristic of acidic soils, and low levels of this family could be considered a factor of soil poverty [64]. Members of the Acidobacteria RB41 clade were also abundant. Recent studies have shown that Acidobacteria RB41 plays a key role in control over the soil carbon cycle [65], and Acidobacteria, in general, are involved in organic and inorganic nitrogen source metabolism [66], and some species can also act as plant growth-promoting rhizobacteria (PGPR) to improve plant growth [67]. At the genus level, other abundant bacteria were the members of the Bacteroidota *Flavobacterium* (3.30%) and *Flavisolibacter* (1.89%) phyla (Figure 2D). Some members of the *Flavobacterium* genus are known producers of β-lactam antibiotics [68]. Other interesting genera, although detected at a rate lower than 2% in the global bacteria community, were members of the Gammaproteobacteria class *Pseudomonas* (1.69%) and *Massilia* (1.28%). Massilia species are commonly associated with plants, and they are proficient in surface colonization, including that of seeds and emerging roots [69]. *Pseudomonas* is a complex genus with many different species exhibiting great metabolic diversity. Consequently, many are able to colonize a wide range of ecological niches. Among these species we can find phytopathogens, plant-growth promoting rhizobacteria, and also biocontrol agents for fungal phytopathogens [70]. Regarding Actinobacteriota, the most represented genera were *Pseudarthrobacter* (1.58%), *Gaiella* (0.70%), *Nocardioides*, and *Streptomyces* (at least 0.70%). Streptomycetales, and particularly the *Streptomyces* genera, which are surprisingly diverse (around 600 species), are responsible for the production of half of all known antibiotics [71] and well-known for their ability to control plant diseases, including soil-borne fungal pathogens [72,73].

### 4.4. Microbial Diversity

Different reports indicate that plant species, or even species genotypes [15,16], tend to assemble relatively distinct rhizobacterial communities [74,75]. In fact, plants exert selective effects on rhizobacterial assemblages in the bulk soil pool to acquire the specific functional traits needed for plant fitness. As a consequence of the strong selective pressure that root plants exert on the microbial communities of the surrounding soils, the microbial diversity in the rhizosphere environment tends to diminish when compared with the microbial diversity of surrounding bulk soils [76]. Interestingly, in our case, healthy soils behaved according to the Shannon index, so that both bacterial and fungal diversities were lower in rhizosphere than in bulk soils (Figure 3C,G). However, in diseased soils, and especially in the case of the fungal community, the observed pattern was exactly the opposite, with diversity increasing in the diseased soils (Figure 3C). A similar result was reported in the infection of wheat by yellow mosaic virus. Indeed, following infection, the alpha diversity of root-associated microbiota significantly increases [77]. We cannot be sure if this increase in fungal diversity in the rhizosphere of diseased plants is caused by the disease or its consequence, although it has been suggested that the exudates from the roots of diseased plants may generate a stress situation in the rhizosphere that destabilizes the fungal community [76]. Otherwise, it could be that the plants had previously suffered an abiotic stress that resulted in an alteration of the secretion pattern in the root system, thus altering the plant-associated microbial community [78].

According to our results, the structure of the microbial communities was affected by both the type of soil and its condition. As seen in the PCoA, in the fungal community, an almost perfect separation of the four types of samples (HR, HB, DR, and DB) is visualized by coordinates 1 and 3. In addition, in the bacterial community, it is possible to visualize a separation between the healthy and diseased ones by looking at coordinates 2 and 3. This pattern is quite similar to that found by Jamil and colleagues [79] when analyzing bacterial communities associated with the Fusarium wilt of banana.

### 4.5. Significantly Different Relative Abundances

The significant differences observed at the level of bacterial taxa between the different soil samples analyzed did not yield particularly striking data, mainly due to the fact that many of the taxa that had significantly different frequencies could not be identified beyond the family level, and thus were identified as uncultured or unclassified bacteria. However, if we look at those bacteria whose abundance increases in the rhizosphere of diseased plants (DR) versus their abundance in the rhizosphere of healthy plants (HR), we see that the four taxa with the lowest LFC values were a nonculturable member of Gemmatimonadaceae (Otu00476), two were members of the order Sphingobacteriales (Otu00385 and Otu01230, the latter identified as *Sphingobacterium*), and one was a member of the Acidobacterium of subgroup 22 (Otu00728). As stated before, *Sphingobacterium* sp. includes species that are environmentally ubiquitous, and some studies have reported the pathogen antagonism and plant growth-promoting potentials of this genus [63]. Gemmatimonadetes have been described to have a putative beneficial effect since their levels are reduced in the rhizosphere of diseased cotton plants [54,80] and olive crops [81]. Thus, we can speculate that the rhizosphere environment of diseased plants could be altered by attracting putative beneficial bacteria [78].

However, identification of fungal taxa with significant differential frequencies in the different soils analyzed yielded interesting results. The comparison of HB and HR soils showed that in the rhizosphere of healthy plants, there is a significant increase in the basidiomycetes (Agaricomycetes) *T. cucumeris* (the teleomorph of *R. solani*) (Otu0116) and *Calyptella* sp. (Otu0163) with respect to the levels observed in HB soils (Supplementary Materials Table S5). *R. solani* is a soil-borne pathogen with a wide host range and which causes diseases in a variety of crops, including agronomical, ornamental, and forestry species, producing root rot in many cases [48]. Although it has a low frequency, *R. solani* can infect hop plants. However, their infection activity is restricted to some particular varieties, like Eureka [82] and Brewer’s Gold [6]. *Calyptella* sp. is also a pathogen that can produce root rot in tomatoes [83], though there is no evidence of its capability to affect hop plants. These results are intriguing, and they could indicate that healthy hop plants are able to maintain these potential pathogens in the rhizosphere without being infected or suffering any significant damage. However, we cannot rule out that these pathogens may contribute in some way to weakening the root system that favors a subsequent infection by *Verticillium* sp., which was much less abundant in the analyzed soils.

When HB and DB soils were compared, we detected that there were increased levels of the basidiomycetes (agaricomycetes) *Ceratobasidium* sp. (Otu0056) and *Mycena* sp. (several OTUs), the zygomycete *Mortierella* sp. (Otu0128), and a member of Glomeralles (Otu0291) in DB soils (Supplementary Materials Table S7). Interestingly, *Ceratobasidium* is a genus closely related to *Thanathephorus*. In fact, *Rhizoctonia* also includes species with teleomorphs in *Ceratobasidium*. Some *Ceratobasidium* species are mycorrhizal symbionts of orchids, and this genus also includes both species that are plant pathogens and the species used as BCAs to control soil-borne plant pathogens, including *R. solani* [84]. The capability of *Mycena* sp. to produce antifungal compounds with different structures such as polyacetylenics, terpenoids, and methoxy acrylates has been described [85,86].

The comparison of HR and DR soils showed that, in HR soils, there was a notable increase in basidiomycetes of the Agaricomycetes group, more specifically, *Calyptella* sp. (Otu0163) and *T. cucumeris (R. solani)* (Otu0116), and other unclassified Auriculariales (Otu0092) and Agarycomycetes (Otu0283). However, in DR, there were significant increases in *Ceratobasidium* sp. (Otu0056), *Mycena* sp. (several OTUs), and some Glomeralles (Otu0291) as the most noteworthy examples (Supplementary Materials Table S8) when compared to HR soils. Genera of Glomeralles are classified as arbuscular mycorrhizal and related fungi (AMF), which are known to contribute to the health status of the host plant by several modes of action, including activation of the defense mechanism against soil-borne pathogens (e.g., *Phytophthora*, *Fusarium*, *Verticillium*) [87]. The highest detected levels of all these putative beneficial microorganisms in the rhizosphere of diseased plants suggests that they could have been recruited to the rhizosphere by the plant upon infection.

The combination of all these data seems to indicate that in the rhizosphere of healthy plants, there are significantly higher levels of putative pathogens like *T. cucumeris* (*R. solani*) and *Calyptella* sp., to which high levels of *Fusarium* sp. should be added. All these pathogens could produce different kinds of injuries to the root system (root rot and cankers), or at least cause some weakening, acting synergistically to somehow favor a later infection of the plant by *Verticillium* sp. through the root system. Upon infection, the plant could trigger some kind of “cry for help” strategy [88], whereby plants subjected to a pathogen-induced stress can recruit beneficial microorganisms to enhance their capability to combat that stress. This fact could explain why, in the rhizosphere of diseased plants, we can find higher levels of beneficial agaricomycetes like *Ceratobasidium* sp. (with antagonistic properties against *R. solani*), *Mycena* sp., and other beneficial fungi such as Glomeralles members. Higher impacts on the composition of fungal microbiota associated with the rhizosphere of diseased plants have also been reported in the case of wilted oil peony (*Paeonia suffruticosa*) [89] and for chili pepper (*Capsicum annuum*) affected by Fusarium wilt disease. In this last manuscript, the authors conclude that fungal communities were less stable and more sensitive to Fusarium wilt disease than bacterial communities [90]. This antagonistic relationship between certain fungal plant pathogens and possible beneficial fungi of the agaricomycete group is interesting and could be related to the fact that hops are a perennial crop, so compared to annual crops, the permanence of the root system in the soil for long times could favor the colonization of slower-growing basidiomycetes. In the future, it could be interesting to check if this situation is typical for this particular plot or if this is what generally occurs with this crop.

### 4.6. Future Prospects

Our findings indicate significant alterations in the rhizosphere of infected plants relative to healthy ones, particularly in the diversity of fungal communities. A potential antagonism between putative beneficial basidiomycetes such as *Mycena* sp. and *Ceratobasidium* sp. on *V. nonalfalfae* and other putative pathogens such as *Calyptella* sp. and *R. solani* merits further investigation. This hypothetical antagonistic effect should be verified in vitro. If confirmed, it may be possible to enhance the colonization of the hop root system by these advantageous fungi, either by establishing new plantations with rhizomes previously inoculated with these microorganisms or by consecutively enriching the crop soil with biofertilizers that contain these advantageous fungi. Alternatively, other putative bacterial BCAs isolated from the rhizosphere environment and selected for their antifungal activity against *V. nonalfalfae* could be explored.

## 5. Conclusions

Bulk soil from a hop farm affected by Verticillium wilt caused by *V. nonalfalfae* in Slovenia exhibited a bacterial composition similar to many other agricultural soils, with a predominance of the Acidobacteria, Actinobacteria, Bacteroidota, Chloroflexi, Gemmatimonadota, Planctomycetota, and Proteobacteria phyla.

At the fungal level, bulk soils were characterized by a high abundance of *Fusarium* sp. (a genus including many plant-pathogenic species and soil-borne fungal pathogen species), which was the dominant taxon in all the soil sample types (healthy and diseased, bulk and rhizosphere) analyzed. However, the relative abundance of *Verticillium* sp. and *V. nonalfalfae* in all samples analyzed was very low, although the levels of the latter increased in the rhizosphere of diseased plants, as determined by quantitative qPCR.

The rhizosphere of diseased plants underwent important changes, with a significant decrease in pathogenic basidiomycetes that can affect the root system, such as *T. cucumeris* (the teleomorph of *R. solani*) and *Calyptella* sp. However, increases in potentially beneficial fungi, such as the basidiomycetes *Ceratobasidium* sp. and *Mycena* sp., the zygomycete *Mortierella* sp., and a member of Glomeralles, were observed. Whether the observed higher relative abundances in putative beneficial fungi, and the decrease in potential fungal pathogens, in the rhizosphere of diseased plants are the cause or consequence of the disease remains to be clarified.

## Figures and Tables

**Table 1:** Physiochemical properties of soil samples collected from healthy and *Verticillium nonalfalfae*-infected plants.

Soil Parameters ^#^	Healthy Plants	Diseased Plants
Granulometric composition	loam	loam
Sand (%)	45.4	47.4
Grind (%)	32.3	30.1
Clay (%)	22.3	22.5
pH value in 1 M KCl/dm^3^	5.7	5.7
Phosphorous Pentoxide–P_2_O_5_ (mg/100g)	30.5	33.2
Potassium oxide–K_2_O (mg/100g)	22.5 ^a^	32.4 ^a^
Magnesium–Mg (mg/100g)	17.0 ^b^	26.8 ^b^
Manganese–Mn (mg/100g)	871.3 ^c^	1341.7 ^c^
Boron–B (mg/kg)	0.814	0.886
Iron–Fe (mg/kg)	23,503.3 ^d^	26,700.0 ^d^
Zinc–Zn (mg/kg)	109.0	105.3
Copper–Cu (mg/kg)	82.6	81.0
Humus (%)	2.8	3.3
Nitrogen-nitrate form HPLC (mg/kg)	55.6 ^e^	144.6 ^e^
Total nitrogen (%)	0.1 ^f^	0.15 ^f^

^#^ Dry matter. ^a–f^ Values marked with the same superscript were significantly different when comparing soils from healthy and diseased plants (*p* ≤ 0.05).

**Table 2:** Estimation of the global effects of soil type and condition on observed richness, Chao1 index, Shannon index, and Pielou’s Evenness indices in the soil samples analyzed. The significance of each factor is indicated with asterisks: ***, *p* < 0.001; **, *p* < 0.01; *, *p* < 0.05; no asterisk, *p* > 0.05.

	Fungi		Bacteria	
	Chi^2^	*p*-Value	Chi^2^	*p*-Value
Observed richness				
Condition	14.780	1.2 × 10^−4^ ***	1.379	0.24
Soil type	2.034	0.154	6.128	0.013 *
Condition: Soil type			7.056	0.008 **
Chao1				
Condition	6.028	0.014 *	1.510	0.219
Soil type	1.32	0.251	7.318	0.007 **
Condition: Soil type			7.212	0.007 **
Shannon				
Condition	1.114	0.291	1.126	0.289
Soil type	1.496	0.221	0.494	0.482
Condition: Soil type	7.241	0.007 **		
Pielou				
Condition	2.656	0.103	0.349	0.555
Soil type	2.403	0.121	0.91867	0.338
Condition: Soil type	8.953	0.003 **		

**Table 3:** Effects of soil type (bulk/rhizosphere) and condition (diseased/healthy) on fungal and bacterial communities. The significance of each factor is indicated with asterisks: ***, *p* < 0.001; *, *p* < 0.05; no asterisk, *p* > 0.05.

	Fungal Community			Bacterial Community		
	Df	F Value	*p*-Value	Df	F Value	*p*-Value
Coordinate 1						
Soil type	1	46.34	1.2 × 10^−5^ ***	1	4.079	0.065
Condition	1	1.97	0.183	1	1.06	0.322
Coordinate 2						
Soil type	1	0.49	0.498	1	19.333	8.7 × 10^−4^ ***
Condition	1	0.50	0.493	1	3.14	0.076
Soil type: Condition	1	7.06	0.021 *	1	5.17	0.049 *
Coordinate 3						
Soil type	1	4.84	0.046 *	1	0.36	0.516
Condition	1	81.91	5.7 × 10^−7^ ***	1	5.92	0.034 *

## Data Availability

Not applicable.

## Supplementary Materials

The following supporting information can be downloaded at: https://www.mdpi.com/article/10.3390/microorganisms11071819/s1. Figure S1: Representative scheme of the Verticillium wilt (*V. nonalfalfae*)-infected hop field sampled for metataxonomic analysis and picture of a plant showing symptoms of Verticillium wilt. Table S1: Fungal OTUs detected in the different soil samples analyzed. Table S2: Bacterial OTUs detected in the different soil samples analyzed. Table S3: Pairwise comparison of estimated marginal means for factors and interactions that significantly affected diversity indices in fungal communities. Table S4: Pairwise comparison of estimated marginal means for factors and interactions that significantly affected diversity indices in bacterial communities. Table S5: Fungal OTUs with significant differential abundances between healthy bulk (HB) and healthy rhizosphere (HR) soils. Table S6: Fungal OTUs with significant differential abundances between diseased bulk (DB) and diseased rhizosphere (DR) soils. Table S7: Fungal OTUs with significant differential abundances between healthy bulk (HB) and diseased bulk (DB) soils. Table S8: Fungal OTUs with significant differential abundances between healthy (HR) and diseased rhizosphere (DR) soils. Table S9: Bacterial OTUs with significant differential abundances between healthy bulk (HB) and healthy rhizosphere (HR) soils. Table S10: Bacterial OTUs with significant differential abundances between diseased bulk (DB) and diseased rhizosphere (DR) soils. Table S11: Bacterial OTUs with significant differential abundances between healthy bulk (HB) and diseased bulk (DB) soil. Table S12: Bacterial OTUs with significant differential abundances between healthy (HR) and diseased rhizosphere (DR) soils.
