# Supplementary material for: Changes in the Microbial Composition of the Rhizosphere of Hop Plants Affected by Verticillium Wilt Caused by Verticillium nonalfalfae
Source: Microorganisms. 2023 Jul 16;11(7):1819. doi: 10.3390/microorganisms11071819 (PMC10385175; doi:10.3390/microorganisms11071819)

Supplementary Material. Figure S1. Representative scheme of the Verticillium wilt (*V. nonalfalfae*) infected hop field sampled for metataxonomic analysis and some important data (Left); and picture of a plant showing symptoms of Verticillium wilt surrounded by healthy plants (Right)

Variety: Celeia
Plant age: 10 years
First record of *V. nonalfalfae: 2020*
Distance between rows: 2.4m
Distance between plants: 1,3m
Location: Savinja valley, Slovenia
GPS: Y 506165,424 X 123975,556
Hop field name: Šentrupert 2-1
Soil and plant sampling: 17.06.2022

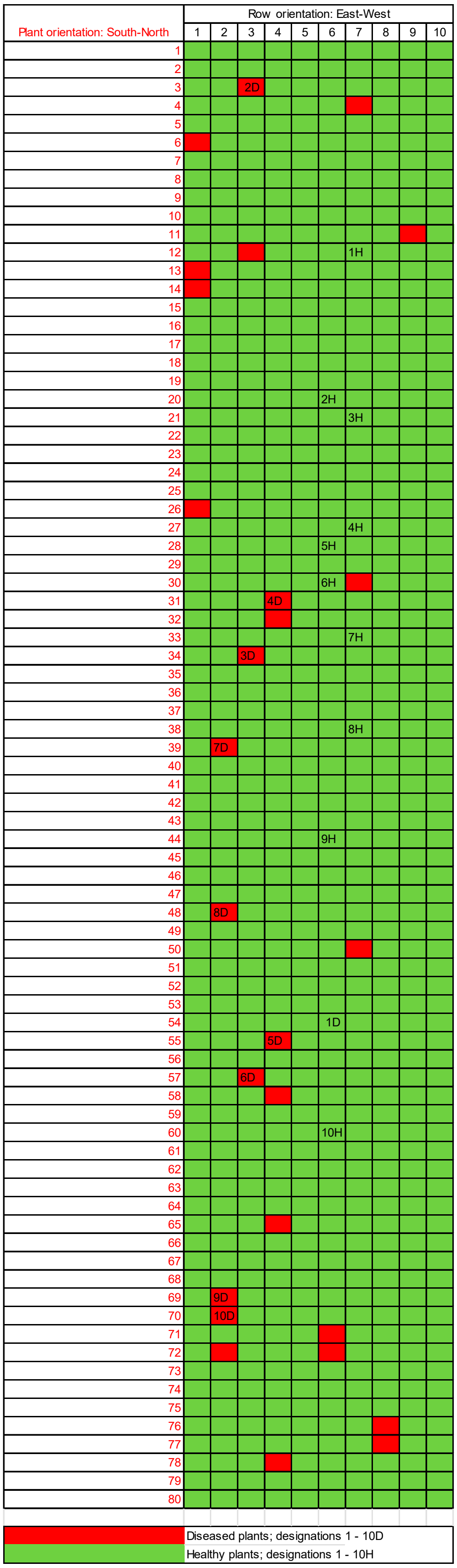

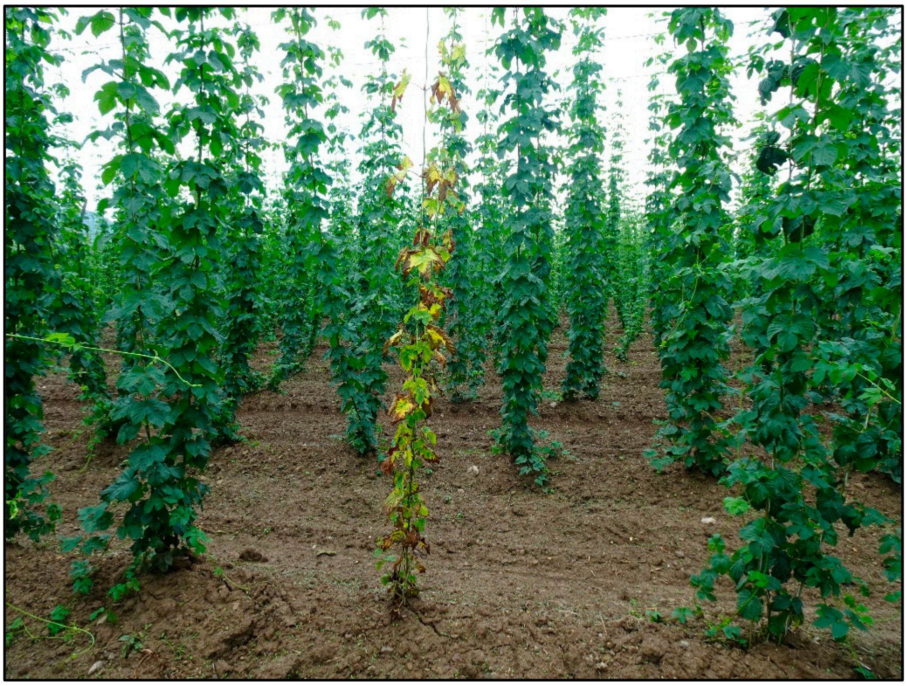

Supplement: Supplementary file 1 [file microorganisms-11-01819-s001.zip › Supplementary Material-Figure S1.pdf]
