# Supplementary material for: Changes in the Microbial Composition of the Rhizosphere of Hop Plants Affected by Verticillium Wilt Caused by Verticillium nonalfalfae
Source: Microorganisms. 2023 Jul 16;11(7):1819. doi: 10.3390/microorganisms11071819 (PMC10385175; doi:10.3390/microorganisms11071819)
Supplement: Supplementary file 1 [file microorganisms-11-01819-s001.zip › Supplementary Material-Table S10.pdf]

Supplementary Material. Table S10.- Bacterial OTUs with significant differential abundances between diseased bulk (DB) and diseased rhizosphere (DR) soils. OTUs with a positive LFC are highlighted on a yellow background while OTUs with a negative LFC are highlighted on a blue background. LFC: log2-fold changes; SE: standard error; *p*-values were adjusted with the False Discovery Rate (FDR) method; unclas.: unclassif.

OTU	LFC	LFC (SE)	Wald statistic	*p*-value	FDR	Phylum	Kingdom	Class	Order	Family	Genus
**Otu01095**	4,624	1,013	4,566	4,97E-06	4,48E-03	Bacteria	Bacteroidota	Bacteroidia	Cytophagales	Cytophagales_unclas.	Cytophagales_unclas.
**Otu00508**	3,053	0,804	3,798	1,46E-04	4,69E-02	Bacteria	Acidobacteriota	Holophagae	Subgroup_7	Subgroup_7_fa	Subgroup_7_ge
**Otu00889**	2,828	0,743	3,807	1,40E-04	4,62E-02	Bacteria	Cyanobacteria	Vampirivibrionia	Vampirovibrionales	Vampirovibrionaceae	Vampirovibrionaceae_ge
**Otu00571**	2,823	0,615	4,587	4,49E-06	4,33E-03	Bacteria	Fibrobacterota	Fibrobacteria	Fibrobacterales	Fibrobacterales_fa	BBMC-4
**Otu00811**	2,732	0,641	4,261	2,04E-05	1,39E-02	Bacteria	Bacteroidota	Bacteroidia	Sphingobacteriales	env,OPS_17	env,OPS_17_ge
**Otu00691**	2,692	0,638	4,220	2,45E-05	1,60E-02	Bacteria	Bacteroidota	Bacteroidia	Sphingobacteriales	env,OPS_17	env,OPS_17_ge
**Otu00224**	2,366	0,367	6,447	1,14E-10	1,87E-06	Bacteria	Bacteria_unclas.	Bacteria_unclas.	Bacteria_unclas.	Bacteria_unclas.	Bacteria_unclas.
**Otu00655**	2,348	0,576	4,076	4,58E-05	2,21E-02	Bacteria	Patescibacteria	Saccharimonadia	Saccharimonadales	Saccharimonadales_fa	Saccharimonadales_ge
**Otu00314**	2,317	0,424	5,468	4,56E-08	1,93E-04	Bacteria	Bacteroidota	Kapabacteria	Kapabacteriales	Kapabacteriales_fa	Kapabacteriales_ge
**Otu00326**	2,290	0,489	4,683	2,82E-06	3,09E-03	Bacteria	Cyanobacteria	Vampirivibrionia	Vampirovibrionales	Vampirovibrionaceae	Vampirovibrionaceae_ge
**Otu00246**	2,167	0,445	4,875	1,09E-06	1,62E-03	Bacteria	Acidobacteriota	Holophagae	Subgroup_7	Subgroup_7_fa	Subgroup_7_ge
**Otu00419**	2,000	0,519	3,857	1,15E-04	4,00E-02	Bacteria	Proteobacteria	Gammaproteobacteria	Pseudomonadales	Pseudohongiellaceae	BIyi10
**Otu00396**	1,957	0,479	4,082	4,46E-05	2,21E-02	Bacteria	Bdellovibrionota	Oligoflexia	0319-6G20	0319-6G20_fa	0319-6G20_ge
**Otu00290**	1,954	0,413	4,730	2,25E-06	2,63E-03	Bacteria	Fibrobacterota	Fibrobacteria	Fibrobacterales	Fibrobacteraceae	possible_genus_04
**Otu00404**	1,942	0,470	4,129	3,65E-05	2,06E-02	Bacteria	Bacteria_unclas.	Bacteria_unclas.	Bacteria_unclas.	Bacteria_unclas.	Bacteria_unclas.
**Otu00291**	1,678	0,372	4,511	6,45E-06	5,28E-03	Bacteria	Bdellovibrionota	Oligoflexia	0319-6G20	0319-6G20_fa	0319-6G20_ge
**Otu00217**	1,674	0,413	4,049	5,13E-05	2,34E-02	Bacteria	Gemmatimonadota	S0134_terrestrial_group	S0134_terrestrial_group_or	S0134_terrestrial_group_fa	S0134_terrestrial_group_ge
**Otu00196**	1,640	0,431	3,807	1,41E-04	4,62E-02	Bacteria	Bacteroidota	Bacteroidia	Cytophagales	Microscillaceae	Ohtaekwangia
**Otu00342**	1,564	0,390	4,012	6,02E-05	2,60E-02	Bacteria	Bacteria_unclas.	Bacteria_unclas.	Bacteria_unclas.	Bacteria_unclas.	Bacteria_unclas.
**Otu00252**	1,513	0,370	4,091	4,30E-05	2,21E-02	Bacteria	Gemmatimonadota	Gemmatimonadetes	Gemmatimonadales	Gemmatimonadaceae	Gemmatimonas
**Otu00253**	1,482	0,373	3,968	7,25E-05	2,83E-02	Bacteria	Bdellovibrionota	Bdellovibrionia	Bacteriovoracales	Bacteriovoracaceae	Peredibacter
**Otu00017**	1,166	0,224	5,202	1,97E-07	5,39E-04	Bacteria	Gemmatimonadota	Gemmatimonadetes	Gemmatimonadales	Gemmatimonadaceae	uncultured
**Otu00057**	1,138	0,272	4,189	2,81E-05	1,77E-02	Bacteria	Bacteroidota	Bacteroidia	Sphingobacteriales	env,OPS_17	env,OPS_17_ge
**Otu00025**	0,995	0,258	3,859	1,14E-04	4,00E-02	Bacteria	Acidobacteriota	Blastocatellia	Blastocatellales	Blastocatellaceae	Blastocatellaceae_unclas.
**Otu00007**	0,910	0,192	4,745	2,09E-06	2,63E-03	Bacteria	Bacteroidota	Bacteroidia	Chitinophagales	Chitinophagaceae	Flavisolibacter
**Otu00105**	-1,312	0,326	-4,020	5,82E-05	2,58E-02	Bacteria	Proteobacteria	Alphaproteobacteria	Reyranellales	Reyranellaceae	Reyranella
**Otu00010**	-1,714	0,376	-4,557	5,19E-06	4,48E-03	Bacteria	Actinobacteriota	Actinobacteria	Micrococcales	Micrococcaceae	Pseudarthrobacter
**Otu00063**	-2,309	0,458	-5,042	4,62E-07	8,41E-04	Bacteria	Proteobacteria	Gammaproteobacteria	Xanthomonadales	Rhodanobacteraceae	Rhodanobacter
**Otu00460**	-2,339	0,573	-4,084	4,42E-05	2,21E-02	Bacteria	Proteobacteria	Alphaproteobacteria	Rhizobiales	Xanthobacteraceae	Rhodopseudomonas
**Otu00334**	-2,556	0,538	-4,753	2,01E-06	2,63E-03	Bacteria	Bacteroidota	Bacteroidia	Cytophagales	Microscillaceae	uncultured
**Otu00723**	-2,766	0,692	-3,997	6,42E-05	2,70E-02	Bacteria	Firmicutes	Bacilli	Bacilli_unclas.	Bacilli_unclas.	Bacilli_unclas.

**Otu01014**	-3,459	0,890	-3,889	1,01E-04	3,75E-02	Bacteria	Verrucomicrobiota	Verrucomicrobiae	Pedosphaerales	Pedosphaeraceae	Pedosphaeraceae_unclas.
**Otu00977**	-3,555	0,811	-4,382	1,17E-05	8,74E-03	Bacteria	Proteobacteria	Gammaproteobacteria	Burkholderiales	Nitrosomonadaceae	GOUTA6
**Otu00472**	-3,570	0,917	-3,895	9,83E-05	3,75E-02	Bacteria	Acidobacteriota	Acidobacteriae	Acidobacteriales	Acidobacteriaceae_(Subgroup_1)	Acidipila-Silvibacterium
**Otu00694**	-3,672	0,903	-4,069	4,72E-05	2,21E-02	Bacteria	Planctomycetota	OM190	OM190_or	OM190_fa	OM190_ge
**Otu00942**	-3,682	0,802	-4,589	4,46E-06	4,33E-03	Bacteria	Verrucomicrobiota	Verrucomicrobiae	Pedosphaerales	Pedosphaeraceae	uncultured
**Otu00271**	-3,780	0,774	-4,886	1,03E-06	1,62E-03	Bacteria	Gemmatimonadota	Gemmatimonadetes	Gemmatimonadales	Gemmatimonadaceae	uncultured
**Otu00609**	-3,788	0,911	-4,160	3,19E-05	1,94E-02	Bacteria	Planctomycetota	Pla4_lineage	Pla4_lineage_or	Pla4_lineage_fa	Pla4_lineage_ge
**Otu00526**	-3,899	0,942	-4,139	3,48E-05	2,04E-02	Bacteria	Proteobacteria	Gammaproteobacteria	Salinisphaerales	Solimonadaceae	Polycyclovorans
**Otu01230**	-3,926	1,017	-3,861	1,13E-04	4,00E-02	Bacteria	Bacteroidota	Bacteroidia	Sphingobacteriales	Sphingobacteriaceae	Sphingobacterium
**Otu00090**	-4,114	0,650	-6,327	2,50E-10	2,05E-06	Bacteria	Verrucomicrobiota	Verrucomicrobiae	Pedosphaerales	Pedosphaeraceae	uncultured
**Otu00222**	-4,352	0,797	-5,460	4,77E-08	1,93E-04	Bacteria	Proteobacteria	Gammaproteobacteria	Xanthomonadales	Xanthomonadaceae	Pseudoxanthomonas
**Otu00476**	-5,098	1,169	-4,359	1,30E-05	9,30E-03	Bacteria	Gemmatimonadota	Gemmatimonadetes	Gemmatimonadales	Gemmatimonadaceae	uncultured
**Otu00444**	-5,164	1,150	-4,489	7,14E-06	5,57E-03	Bacteria	Patescibacteria	Saccharimonadia	Saccharimonadales	Saccharimonadaceae	Saccharimonadaceae_unclas.
**Otu00761**	-5,170	1,267	-4,081	4,48E-05	2,21E-02	Bacteria	Patescibacteria	Saccharimonadia	Saccharimonadales	Saccharimonadales_fa	Saccharimonadales_ge
**Otu00897**	-5,508	1,381	-3,989	6,63E-05	2,72E-02	Bacteria	Planctomycetota	Pla4_lineage	Pla4_lineage_or	Pla4_lineage_fa	Pla4_lineage_ge
**Otu01466**	-5,902	1,486	-3,972	7,13E-05	2,83E-02	Bacteria	Patescibacteria	Saccharimonadia	Saccharimonadales	Saccharimonadales_unclas.	Saccharimonadales_unclas.
**Otu00416**	-5,963	1,155	-5,161	2,45E-07	5,74E-04	Bacteria	Acidobacteriota	Holophagae	Holophagales	Holophagaceae	Holophaga
**Otu00569**	-6,209	1,145	-5,422	5,90E-08	1,93E-04	Bacteria	Verrucomicrobiota	Verrucomicrobiae	Pedosphaerales	Pedosphaeraceae	uncultured
**Otu00666**	-6,901	1,367	-5,050	4,42E-07	8,41E-04	Bacteria	FCPU426	FCPU426_cl	FCPU426_or	FCPU426_fa	FCPU426_ge
**Otu00442**	-7,119	1,866	-3,816	1,36E-04	4,62E-02	Bacteria	Bacteroidota	Bacteroidia	Sphingobacteriales	env,OPS_17	env,OPS_17_ge
