# Supplementary material for: Changes in the Microbial Composition of the Rhizosphere of Hop Plants Affected by Verticillium Wilt Caused by Verticillium nonalfalfae
Source: Microorganisms. 2023 Jul 16;11(7):1819. doi: 10.3390/microorganisms11071819 (PMC10385175; doi:10.3390/microorganisms11071819)
Supplement: Supplementary file 1 [file microorganisms-11-01819-s001.zip › Supplementary Material-Table S11.pdf]

Supplementary Material. Table S11.- Bacterial OTUs with significant differential abundances between healthy bulk (HB) and diseased bulk (DB) soils. OTUs with a positive LFC are highlighted on a yellow background while OTUs with a negative LFC are highlighted on a blue background. LFC: log2-fold changes; SE: standard error; *p*-values were adjusted with the False Discovery Rate (FDR) method; unclas.: unclassified

	LFC	LFC (SE)	Wald statistic	*p*-value	FDR	Phylum	Kingdom	Class	Order	Family	Genus
**Otu01429**	5,306	1,179	4,501	6,77E-06	0,027	Bacteria	Bacteria_unclas.	Bacteria_unclas.	Bacteria_unclas.	Bacteria_unclas.	Bacteria_unclas.
**Otu00893**	3,841	0,786	4,885	1,04E-06	0,008	Bacteria	Patescibacteria	Saccharimonadia	Saccharimonadales	Saccharimonadales_unclas.	Saccharimonadales_unclas.
**Otu00097**	1,305	0,242	5,400	6,67E-08	0,001	Bacteria	Chloroflexi	Ktedonobacteria	C0119	C0119_fa	C0119_ge
**Otu01273**	-5,891	1,310	-4,496	6,93E-06	0,027	Bacteria	Chloroflexi	Anaerolineae	Anaerolineales	Anaerolineaceae	uncultured
