# Supplementary material for: Changes in the Microbial Composition of the Rhizosphere of Hop Plants Affected by Verticillium Wilt Caused by Verticillium nonalfalfae
Source: Microorganisms. 2023 Jul 16;11(7):1819. doi: 10.3390/microorganisms11071819 (PMC10385175; doi:10.3390/microorganisms11071819)
Supplement: Supplementary file 1 [file microorganisms-11-01819-s001.zip › Supplementary Material-Table S12.pdf]

Supplementary Material. Table S12.- Bacterial OTUs with significant differencial abundances between healthy (HR) and diseased rizhosphere (DR) soils. OTUs with a positive LFC are highlighted on a yellow background while OTUs with a negative LFC are highlighted on a blue background. LFC: log2-fold changes; SE: standard error; *p*-values were adjusted with the False Discovery Rate (FDR) method; unclas.: unclassified

OTU	LFC	LFC (SE)	Wald statistic	*p*-value	FDR	Phylum	Class	Order	Family	Genus	Species
**Otu01133**	6,613	1,365	4,843	1,28E-06	2,28E-03	Bacteria	Bacteroidota	Bacteroidia	Chitinophagales	Chitinophagaceae	Chitinophaga
**Otu00715**	3,561	0,876	4,065	4,80E-05	2,96E-02	Bacteria	Cyanobacteria	Cyanobacteriia	Cyanobacteriales	Nostocaceae	Nostoc_PCC-73102
**Otu00508**	3,121	0,783	3,987	6,68E-05	3,83E-02	Bacteria	Acidobacteriota	Holophagae	Subgroup_7	Subgroup_7_fa	Subgroup_7_ge
**Otu00673**	2,539	0,587	4,327	1,51E-05	1,40E-02	Bacteria	Bacteria_unclas.	Bacteria_unclas.	Bacteria_unclas.	Bacteria_unclas.	Bacteria_unclas.
**Otu00352**	2,356	0,482	4,894	9,88E-07	2,27E-03	Bacteria	Planctomycetota	Phycisphaerae	Tepidisphaerales	WD2101_soil_group	WD2101_soil_group_ge
**Otu00246**	1,959	0,376	5,211	1,88E-07	6,03E-04	Bacteria	Acidobacteriota	Holophagae	Subgroup_7	Subgroup_7_fa	Subgroup_7_ge
**Otu00040**	1,799	0,408	4,41	1,03E-05	1,15E-02	Bacteria	Bacteria_unclas.	Bacteria_unclas.	Bacteria_unclas.	Bacteria_unclas.	Bacteria_unclas.
**Otu00097**	1,458	0,338	4,307	1,66E-05	1,40E-02	Bacteria	Chloroflexi	Ktedonobacteria	C0119	C0119_fa	C0119_ge
**Otu00017**	1,324	0,233	5,696	1,23E-08	9,84E-05	Bacteria	Gemmatimonadota	Gemmatimonadetes	Gemmatimonadales	Gemmatimonadaceae	uncultured
**Otu00014**	1,292	0,312	4,142	3,44E-05	2,40E-02	Bacteria	Proteobacteria	Gammaproteobacteria	Burkholderiales	Oxalobacteraceae	Massilia
**Otu00195**	1,234	0,299	4,123	3,74E-05	2,50E-02	Bacteria	Chloroflexi	Chloroflexia	Chloroflexales	Roseiflexaceae	uncultured
**Otu00118**	1,092	0,26	4,208	2,57E-05	1,87E-02	Bacteria	Planctomycetota	Phycisphaerae	Tepidisphaerales	WD2101_soil_group	WD2101_soil_group_ge
**Otu00007**	0,715	0,182	3,919	8,91E-05	4,61E-02	Bacteria	Bacteroidota	Bacteroidia	Chitinophagales	Chitinophagaceae	Flavisolibacter
**Otu00263**	-1,885	0,462	-4,077	4,56E-05	2,93E-02	Bacteria	Proteobacteria	Gammaproteobacteria	Burkholderiales	Nitrosomonadaceae	MND1
**Otu00163**	-2,411	0,375	-6,432	1,26E-10	2,02E-06	Bacteria	Proteobacteria	Gammaproteobacteria	Burkholderiales	TRA3-20	TRA3-20_ge
**Otu00117**	-2,419	0,55	-4,399	1,09E-05	1,15E-02	Bacteria	Verrucomicrobiota	Verrucomicrobiae	Opitutales	Opitutaceae	Lacunisphaera
**Otu00164**	-2,531	0,564	-4,492	7,07E-06	9,20E-03	Bacteria	Bacteroidota	Bacteroidia	Cytophagales	Microscillaceae	Microscillaceae_unclas.
**Otu00526**	-2,742	0,698	-3,929	8,54E-05	4,57E-02	Bacteria	Proteobacteria	Gammaproteobacteria	Salinisphaerales	Solimonadaceae	Polycyclovorans
**Otu00271**	-2,78	0,528	-5,27	1,36E-07	5,46E-04	Bacteria	Gemmatimonadota	Gemmatimonadetes	Gemmatimonadales	Gemmatimonadaceae	uncultured
**Otu00090**	-2,841	0,569	-4,995	5,90E-07	1,58E-03	Bacteria	Verrucomicrobiota	Verrucomicrobiae	Pedosphaerales	Pedosphaeraceae	uncultured
**Otu00826**	-2,933	0,728	-4,027	5,64E-05	3,35E-02	Bacteria	Myxococcota	Polyangia	Polyangiales	BIrii41	BIrii41_ge
**Otu00151**	-3,079	0,58	-5,305	1,12E-07	5,46E-04	Bacteria	Bacteroidota	Bacteroidia	Cytophagales	Microscillaceae	Hassallia
**Otu00942**	-3,097	0,68	-4,557	5,18E-06	7,56E-03	Bacteria	Verrucomicrobiota	Verrucomicrobiae	Pedosphaerales	Pedosphaeraceae	uncultured
**Otu00609**	-3,248	0,76	-4,271	1,94E-05	1,56E-02	Bacteria	Planctomycetota	Pla4_lineage	Pla4_lineage_or	Pla4_lineage_fa	Pla4_lineage_ge
**Otu00512**	-3,313	0,755	-4,387	1,15E-05	1,15E-02	Bacteria	Planctomycetota	OM190	OM190_or	OM190_fa	OM190_ge
**Otu00666**	-3,731	0,945	-3,949	7,85E-05	4,34E-02	Bacteria	FCPU426	FCPU426_cl	FCPU426_or	FCPU426_fa	FCPU426_ge
**Otu00977**	-3,97	0,824	-4,817	1,46E-06	2,34E-03	Bacteria	Proteobacteria	Gammaproteobacteria	Burkholderiales	Nitrosomonadaceae	GOUTA6
**Otu00385**	-4,275	0,954	-4,48	7,46E-06	9,20E-03	Bacteria	Bacteroidota	Bacteroidia	Sphingobacteriales	NS11-12_marine_group	NS11-12_marine_group_ge
**Otu00728**	-4,426	1,028	-4,307	1,66E-05	1,40E-02	Bacteria	Acidobacteriota	Subgroup_22	Subgroup_22_or	Subgroup_22_fa	Subgroup_22_ge
**Otu01230**	-5,248	1,238	-4,238	2,25E-05	1,72E-02	Bacteria	Bacteroidota	Bacteroidia	Sphingobacteriales	Sphingobacteriaceae	Sphingobacterium
**Otu00476**	-5,513	1,136	-4,852	1,22E-06	2,28E-03	Bacteria	Gemmatimonadota	Gemmatimonadetes	Gemmatimonadales	Gemmatimonadaceae	uncultured
