# Supplementary material for: Changes in the Microbial Composition of the Rhizosphere of Hop Plants Affected by Verticillium Wilt Caused by Verticillium nonalfalfae
Source: Microorganisms. 2023 Jul 16;11(7):1819. doi: 10.3390/microorganisms11071819 (PMC10385175; doi:10.3390/microorganisms11071819)
Supplement: Supplementary file 1 [file microorganisms-11-01819-s001.zip › Supplementary Material-Table S3.pdf]

Supplementary Material. Table S3. Pairwise comparison of estimated marginal means for factors and interactions that significantly affected diversity indices in fungal communities (see Main text). *p*-values were adjusted with FDR method. The significance of each factor is indicated with asterisks: ***: $p < 0.001$; *: $p < 0.05$.

D: diseased condition; H: healthy condition; DB: diseased bulk soil; HB: healthy bulk soil; DR: diseased rhizosphere soil; HR: healthy rhizosphere soil. SE: Standart Error; df: degrees of freedom; Inf: infinite

	Estimate	SE	df	*z*-ratio	*p*-value
**Observed richness**					
**D – H**	0.094	0.0245	Inf	3844	0.0001***
**Chao1 Index**					
**D – H**	0.0906	0.0369	13	2456	0.0289*
**Shannon index**					
**D R - H R**	0.09904887	0.03601582	12	2.750149	0.04796495*
**D B - H B**	-0.03802168	0.03601582	12	-1.055694	0.31189936
**H B - H R**	0.09301458	0.03601582	12	2.582603	0.04796495*
**D B - D R**	-0.04405596	0.03601582	12	-1.223239	0.31189936
**Pielou index**					
**D R - H R**	0.08835823	0.03395578	12	2.602156	0.04626758*
**D B - H B**	-0.05534042	0.03395578	12	-1.629779	0.14705405
**H B - H R**	0.09106042	0.03395578	12	2.681735	0.04626758*
**D B - D R**	-0.05263824	0.03395578	12	-1.550200	0.14705405
