# Supplementary material for: Changes in the Microbial Composition of the Rhizosphere of Hop Plants Affected by Verticillium Wilt Caused by Verticillium nonalfalfae
Source: Microorganisms. 2023 Jul 16;11(7):1819. doi: 10.3390/microorganisms11071819 (PMC10385175; doi:10.3390/microorganisms11071819)
Supplement: Supplementary file 1 [file microorganisms-11-01819-s001.zip › Supplementary Material-Table S4.pdf]

Supplementary Material. Table S4. Pairwise comparison of estimated marginal means for factors and interactions that significantly affected diversity indices in bacterial communities (see Main text). *p*-values were adjusted with FDR method. The significance of each factor is indicated with asterisks: *: $p < 0.05$.

D: diseased condition; H: healthy condition; DB: diseased bulk soil; HB: healthy bulk soil; DR: diseased rhizosphere soil; HR: healthy rhizosphere soil. SE: Standart Error; df: degrees of freedom; Inf: infinite

	**Estimation**	**SE**	**df**	***z*-ratio**	***p*-value**
**Observed richness**					
**D R - H R**	0.266	0.102	Inf	2.592	0.026*
**D B - H B**	-0.120	0.102	Inf	-1.173	0.241
**H B - H R**	0.131	0.102	Inf	1.278	0.241
**D B - D R**	-0.255	0.102	Inf	-2.487	0.026*
**Chao1 index**					
**D R - H R**	0.305	0.119	12	2.573	0.049*
**D B - H B**	-0.146	0.119	12	-1.229	0.297
**H B - H R**	0.129	0.119	12	1.091	0.297
**D B - D R**	-0.322	0.119	12	-2.711	0.049
**Shannon index**					
**D R - H R**	0.017	0.016	13	1.061	0.495
**D B - H B**	0.017	0.016	13	1.061	0.495
**H B - H R**	0.012	0.016	13	0.703	0.495
**D B - D R**	0.012	0.016	13	0.703	0.495
