# Supplementary material for: Changes in the Microbial Composition of the Rhizosphere of Hop Plants Affected by Verticillium Wilt Caused by Verticillium nonalfalfae
Source: Microorganisms. 2023 Jul 16;11(7):1819. doi: 10.3390/microorganisms11071819 (PMC10385175; doi:10.3390/microorganisms11071819)
Supplement: Supplementary file 1 [file microorganisms-11-01819-s001.zip › Supplementary Material-Table S5.pdf]

Supplementary Material. Table S5.- Fungal OTUs with significant differential abundances between healthy bulk (HB) and healthy rhizosphere (HR) soils. OTUs with a positive LFC are highlighted on a yellow background while OTUs with a negative LFC are highlighted on a blue background. LFC: log2-fold changes; SE: standard error; *p*-values were adjusted with the False Discovery Rate (FDR) method; unclas.: unclassified

OTU	LFC	LFC (SE)	Wald statistic	*p*-value	FDR	Phylum	Class	Order	Family	Genus	Species
**Otu0032**	7,381	1,952	3,782	1.6e-04	1.8e-02	Basidiomycota	Agaricomycetes	Agaricales	Entolomataceae	Entoloma	Entoloma unclas.
**Otu0106**	6,335	1,841	3,44	5.8e-04	4.7e-02	Chytridiomycota	Chytridiomycetes	Spizellomycetales	Spizellomycetaceae	Spizellomycetaceae unclas.	Spizellomycetaceae unclas.
**Otu0081**	2,302	0,637	3,614	3.0e-04	2.7e-02	Fungi unclas.	Fungi unclas.	Fungi unclas.	Fungi unclas.	Fungi unclas.	Fungi unclas.
**Otu0034**	1,898	0,478	3,968	7.2e-05	9.5e-03	Ascomycota	Sordariomycetes	Sordariomycetes unclas.	Sordariomycetes unclas.	Sordariomycetes unclas.	Sordariomycetes unclas.
**Otu0051**	-3,028	0,826	-3,668	2.4e-04	2.3e-02	Ascomycota	Dothideomycetes	Pleosporales	Didymosphaeriaceae	Roussoella	Roussoella japanensis
**Otu0088**	-4,146	1,101	-3,767	1.7e-04	1.8e-02	Ascomycota	Ascomycota unclas.	Ascomycota unclas.	Ascomycota unclas.	Ascomycota unclas.	Ascomycota unclas.
**Otu0165**	-4,985	1,246	-4	6.3e-05	9.0e-03	Chytridiomycota	Chytridiomycetes	Rhizophlyctidales	Rhizophlyctidaceae	Rhizophlyctis	Rhizophlyctis rosea
**Otu0234**	-5,149	1,283	-4,012	6.0e-05	9.0e-03	Fungi unclas.	Fungi unclas.	Fungi unclas.	Fungi unclas.	Fungi unclas.	Fungi unclas.
**Otu0006**	-5,153	1,495	-3,446	5.7e-04	4.7e-02	Fungi unclas.	Fungi unclas.	Fungi unclas.	Fungi unclas.	Fungi unclas.	Fungi unclas.
**Otu0262**	-5,418	1,2	-4,516	6.3e-06	1.1e-03	Fungi unclas.	Fungi unclas.	Fungi unclas.	Fungi unclas.	Fungi unclas.	Fungi unclas.
**Otu0410**	-6,175	1,571	-3,931	8.5e-05	1.0e-02	Ascomycota	Sordariomycetes	Diaporthales	Togniniaceae	Phaeoacremonium	Phaeoacremonium tuscanum
**Otu0394**	-6,684	1,797	-3,719	2.0e-04	2.0e-02	Basidiomycota	Agaricomycetes	Russulales	Stephanosporaceae	unclas. Stephanosporaceae	Stephanosporaceae sp
**Otu0050**	-7,098	0,729	-9,733	2.2e-22	3.7e-19	Ascomycota	Sordariomycetes	Hypocreales	Cordycipitaceae	Cordycipitaceae unclas.	Cordycipitaceae unclas.
**Otu0080**	-9,947	1,152	-8,634	5.9e-18	5.1e-15	Ascomycota	Ascomycota unclas.	Ascomycota unclas.	Ascomycota unclas.	Ascomycota unclas.	Ascomycota unclas.
**Otu0460**	-19,96	3,388	-5,891	3.8e-09	7.3e-07	Ascomycota	Eurotiomycetes	Eurotiales	Trichocomaceae	Paecilomyces	Paecilomyces penicillatus
**Otu0398**	-21,017	3,387	-6,206	5.4e-10	1.2e-07	Zygomycota	Zygomycota class Incertae sedis	Mortierellales	Mortierellaceae	Mortierella	Mortierella unclas.
**Otu0339**	-21,327	3,386	-6,299	3.0e-10	7.3e-08	Fungi unclas.	Fungi unclas.	Fungi unclas.	Fungi unclas.	Fungi unclas.	Fungi unclas.
**Otu0283**	-21,828	3,385	-6,448	1.1e-10	3.2e-08	Basidiomycota	Agaricomycetes	Agaricomycetes unclas.	Agaricomycetes unclas.	Agaricomycetes unclas.	Agaricomycetes unclas.
**Otu0163**	-23,557	3,384	-6,961	3.4e-12	1.2e-09	Basidiomycota	Agaricomycetes	Agaricales	Marasmiaceae	Calyptella	Calyptella sp.
**Otu0116**	-23,973	3,384	-7,085	1.4e-12	6.0e-10	Basidiomycota	Agaricomycetes	Cantharellales	Ceratobasidiaceae	Thanatephorus	Thanatephorus cucumeris
**Otu0111**	-24,053	3,384	-7,108	1.2e-12	6.0e-10	Fungi unclas.	Fungi unclas.	Fungi unclas.	Fungi unclas.	Fungi unclas.	Fungi unclas.
