# Supplementary material for: Changes in the Microbial Composition of the Rhizosphere of Hop Plants Affected by Verticillium Wilt Caused by Verticillium nonalfalfae
Source: Microorganisms. 2023 Jul 16;11(7):1819. doi: 10.3390/microorganisms11071819 (PMC10385175; doi:10.3390/microorganisms11071819)
Supplement: Supplementary file 1 [file microorganisms-11-01819-s001.zip › Supplementary Material-Table S6.pdf]

Supplementary Material. Table S6.- Fungal OTUs with significant differential abundances between diseased bulk (DB) and diseased rhizosphere (DR) soils. OTUs with a positive LFC are highlighted on a yellow background while OTUs with a negative LFC are highlighted on a blue background. LFC: log2-fold changes; SE: standard error; *p*-values were adjusted with the False Discovery Rate (FDR) method; unclas.: unclassified

OTU	LFC	LFC (SE)	Wald statistic	*p*-value	FDR	Phylum	Class	Order	Family	Genus	Species
**Otu0159**	22,511	3,384	6,652	2.9e-11	2.7e-08	Chytridiomycota	Chytridiomycetes	Spizellomycetales	Spizellomycetaceae	Powellomyces	Powellomyces sp
**Otu0224**	21,919	3,385	6,476	9.4e-11	5.9e-08	Ascomycota	Ascomycota unclas.	Ascomycota unclas.	Ascomycota unclas.	Ascomycota unclas.	Ascomycota unclas.
**Otu0228**	21,454	3,385	6,338	2.3e-10	8.7e-08	Zygomycota	Zygomycota class Incertae sedis	Mortierellales	Mortierellaceae	Mortierella	Mortierella sp
**Otu0211**	20,843	3,386	6,155	7.5e-10	2.3e-07	Ascomycota	Sordariomycetes	Sordariales	Lasiosphaeriaceae	Lasiosphaeris	Lasiosphaeris hispida
**Otu0110**	-3,825	0,963	-3,97	7.2e-05	1.2e-02	Fungi unclas.	Fungi unclas.	Fungi unclas.	Fungi unclas.	Fungi unclas.	Fungi unclas.
**Otu0099**	-3,976	0,926	-4,293	1.8e-05	4.1e-03	Basidiomycota	Tremellomycetes	Tremellales	Tremellales family Incertae sedis	Cryptococcus	Cryptococcus podzolicus
**Otu0248**	-5,977	1,418	-4,215	2.5e-05	5.2e-03	Ascomycota	Sordariomycetes	Sordariales	Lasiosphaeriaceae	Cladorrhinum	Cladorrhinum sp
**Otu0287**	-6,268	1,503	-4,171	3.0e-05	5.6e-03	Basidiomycota	Microbotryomycetes	Sporidiobolales	Sporidiobolales family Incertae sedis	Sporidiobolales family Incertae sedis unclas.	Sporidiobolales family Incertae sedis unclas.
**Otu0335**	-6,508	1,802	-3,611	3.0e-04	4.7e-02	Fungi unclas.	Fungi unclas.	Fungi unclas.	Fungi unclas.	Fungi unclas.	Fungi unclas.
**Otu0139**	-9,25	1,55	-5,97	2.4e-09	6.3e-07	Chytridiomycota	Chytridiomycetes	Rhizophydiales	Rhizophydiales family Incertae sedis	Operculomyces	Operculomyces laminatus
**Otu0358**	-21,522	3,387	-6,354	2.1e-10	8.7e-08	Ascomycota	Sordariomycetes	Xylariales	Xylariaceae	Xylaria	Xylaria unclas.
**Otu0246**	-22,687	3,385	-6,702	2.1e-11	2.7e-08	Fungi unclas.	Fungi unclas.	Fungi unclas.	Fungi unclas.	Fungi unclas.	Fungi unclas.
