# Supplementary material for: Changes in the Microbial Composition of the Rhizosphere of Hop Plants Affected by Verticillium Wilt Caused by Verticillium nonalfalfae
Source: Microorganisms. 2023 Jul 16;11(7):1819. doi: 10.3390/microorganisms11071819 (PMC10385175; doi:10.3390/microorganisms11071819)
Supplement: Supplementary file 1 [file microorganisms-11-01819-s001.zip › Supplementary Material-Table S7.pdf]

Supplementary Material. Table S7.- Fungal OTUs with significant differential abundances between healthy bulk (HB) and diseased bulk (DB) soils. OTUs with a positive LFC are highlighted on a yellow background while OTUs with a negative LFC are highlighted on a blue background. LFC: log2-fold changes; SE: standard error; *p*-values were adjusted with the False Discovery Rate (FDR) method; unclas.: unclassified

OTU	LFC	LFC (SE)	Wald statistic	*p*-value	FDR	Phylum	Class	Order	Family	Genus	Species
**Otu0267**	21,978	3,386	6,492	8.5e-11	1.7e-08	Ascomycota	Ascomycota unclas.	Ascomycota unclas.	Ascomycota unclas.	Ascomycota unclas.	Ascomycota unclas.
**Otu0065**	7,691	1,862	4,131	3.6e-05	2.7e-03	Basidiomycota	Agaricomycetes	Agaricales	Agaricales unclas.	Agaricales unclas.	Agaricales unclas.
**Otu0300**	6,63	1,977	3,354	8.0e-04	4.1e-02	Ascomycota	Dothideomycetes	Pleosporales	Sporormiaceae	Preussia	Preussia unclas.
**Otu0087**	4,595	1,125	4,086	4.4e-05	3.0e-03	Fungi unclas.	Fungi unclas.	Fungi unclas.	Fungi unclas.	Fungi unclas.	Fungi unclas.
**Otu0037**	3,632	0,967	3,757	1.7e-04	1.1e-02	Fungi unclas.	Fungi unclas.	Fungi unclas.	Fungi unclas.	Fungi unclas.	Fungi unclas.
**Otu0013**	2,108	0,608	3,466	5.3e-04	2.9e-02	Ascomycota	Dothideomycetes	Pleosporales	Pleosporaceae	Alternaria	Alternaria porri
**Otu0003**	-1,763	0,43	-4,1	4.1e-05	3.0e-03	Ascomycota	Dothideomycetes	Pleosporales	Pleosporales family Incertae sedis	Pleosporales family Incertae sedis unclas.	Pleosporales family Incertae sedis unclas.
**Otu0033**	-2,248	0,647	-3,477	5.1e-04	2.9e-02	Ascomycota	Dothideomycetes	Pleosporales	Pleosporales unclas.	Pleosporales unclas.	Pleosporales unclas.
**Otu0027**	-3,055	0,78	-3,916	9.0e-05	5.9e-03	Zygomycota	Zygomycota class Incertae sedis	Mortierellales	Mortierellaceae	Mortierella	Mortierella unclas.
**Otu0141**	-3,095	0,916	-3,38	7.2e-04	3.9e-02	Ascomycota	Leotiomycetes	Leotiomycetes unclas.	Leotiomycetes unclas.	Leotiomycetes unclas.	Leotiomycetes unclas.
**Otu0071**	-3,476	0,755	-4,601	4.2e-06	3.7e-04	Basidiomycota	Tremellomycetes	Holtermanniales	Holtermanniales family Incertae sedis	Holtermannia	Holtermannia corniformis
**Otu0102**	-3,771	0,865	-4,359	1.3e-05	1.1e-03	Ascomycota	Dothideomycetes	Pleosporales	Pleosporales unclas.	Pleosporales unclas.	Pleosporales unclas.
**Otu0006**	-4,391	1,154	-3,805	1.4e-04	9.0e-03	Fungi unclas.	Fungi unclas.	Fungi unclas.	Fungi unclas.	Fungi unclas.	Fungi unclas.
**Otu0382**	-4,992	1,514	-3,296	9.8e-04	5.0e-02	Ascomycota	Dothideomycetes	Dothideomycetes unclas.	Dothideomycetes unclas.	Dothideomycetes unclas.	Dothideomycetes unclas.
**Otu0179**	-5,844	1,235	-4,734	2.2e-06	2.0e-04	Ascomycota	Sordariomycetes	Hypocreales	Hypocreales family Incertae sedis	Acremonium	Acremonium nepalense
**Otu0109**	-6,233	1,261	-4,944	7.7e-07	7.4e-05	Ascomycota	Sordariomycetes	Sordariomycetes unclas.	Sordariomycetes unclas.	Sordariomycetes unclas.	Sordariomycetes unclas.
**Otu0050**	-6,3	0,897	-7,021	2.2e-12	6.4e-10	Ascomycota	Sordariomycetes	Hypocreales	Cordycipitaceae	Cordycipitaceae unclas.	Cordycipitaceae unclas.
**Otu0234**	-6,852	1,842	-3,72	2.0e-04	1.2e-02	Fungi unclas.	Fungi unclas.	Fungi unclas.	Fungi unclas.	Fungi unclas.	Fungi unclas.
**Otu0265**	-6,922	1,926	-3,595	3.2e-04	1.9e-02	Basidiomycota	Agaricomycetes	Auriculariales	Auriculariales unclas.	Auriculariales unclas.	Auriculariales unclas.
**Otu0403**	-6,994	1,643	-4,258	2.1e-05	1.7e-03	Fungi unclas.	Fungi unclas.	Fungi unclas.	Fungi unclas.	Fungi unclas.	Fungi unclas.
**Otu0352**	-7,528	1,822	-4,131	3.6e-05	2.7e-03	Basidiomycota	Tremellomycetes	Trichosporonales	Trichosporonaceae	Trichosporon	Trichosporon unclas.
**Otu0316**	-7,546	1,846	-4,087	4.4e-05	3.0e-03	Basidiomycota	Basidiomycota unclas.	Basidiomycota unclas.	Basidiomycota unclas.	Basidiomycota unclas.	Basidiomycota unclas.
**Otu0097**	-9,234	1,622	-5,695	1.2e-08	1.3e-06	Fungi unclas.	Fungi unclas.	Fungi unclas.	Fungi unclas.	Fungi unclas.	Fungi unclas.
**Otu0088**	-9,243	1,196	-7,728	1.1e-14	5.5e-12	Ascomycota	Ascomycota unclas.	Ascomycota unclas.	Ascomycota unclas.	Ascomycota unclas.	Ascomycota unclas.
**Otu0080**	-10,453	1,283	-8,146	3.8e-16	3.8e-13	Ascomycota	Ascomycota unclas.	Ascomycota unclas.	Ascomycota unclas.	Ascomycota unclas.	Ascomycota unclas.
**Otu0423**	-20,145	2,517	-8,004	1.2e-15	8.1e-13	Ascomycota	Ascomycota unclas.	Ascomycota unclas.	Ascomycota unclas.	Ascomycota unclas.	Ascomycota unclas.

**Otu0468**	-20,425	2,474	-8,257	1.5e-16	3.0e-13	Basidiomycota	Agaricomycetes	Agaricomycetes unclas.	Agaricomycetes unclas.	Agaricomycetes unclas.	Agaricomycetes unclas.
**Otu0531**	-20,505	3,387	-6,053	1.4e-09	1.5e-07	Chytridiomycota	Chytridiomycetes	Rhizophlyctidales	Rhizophlyctidaceae	Rhizophlyctis	Rhizophlyctis rosea
**Otu0493**	-20,677	3,387	-6,105	1.0e-09	1.2e-07	Basidiomycota	Agaricomycetes	Agaricales	Mycenaceae	Mycena	Mycena unclas.
**Otu0453**	-21,017	3,387	-6,102	1.1e-09	1.2e-07	Basidiomycota	Agaricomycetes	Agaricales	Mycenaceae	Mycena	Mycena unclas.
**Otu0308**	-21,096	3,386	-6,23	4.7e-10	5.9e-08	Ascomycota	Ascomycota unclas.	Ascomycota unclas.	Ascomycota unclas.	Ascomycota unclas.	Ascomycota unclas.
**Otu0344**	-21,327	3,386	-6,321	2.6e-10	3.5e-08	Basidiomycota	Agaricomycetes	Agaricales	Mycenaceae	Mycena	Mycena unclas.
**Otu0173**	-21,443	3,384	-6,336	2.4e-10	3.4e-08	Chytridiomycota	Chytridiomycetes	Spizellomycetales	Spizellomycetaceae	Spizellomyces	Spizellomyces unclas.
**Otu0311**	-21,453	3,385	-6,337	2.3e-10	3.4e-08	Ascomycota	Orbiliomycetes	Orbiliales	Orbiliaceae	Orbiliaceae unclas.	Orbiliaceae unclas.
**Otu0233**	-21,85	3,385	-6,455	1.1e-10	1.8e-08	Chytridiomycota	Chytridiomycetes	Spizellomycetales	Spizellomycetaceae	Spizellomycetaceae unclas.	Spizellomycetaceae unclas.
**Otu0291**	-21,871	3,385	-6,461	1.0e-10	1.8e-08	Glomeromycota	Glomeromycetes	Glomerales	Glomerales unclas.	Glomerales unclas.	Glomerales unclas.
**Otu0182**	-22,411	3,384	-6,622	3.5e-11	8.0e-09	Fungi unclas.	Fungi unclas.	Fungi unclas.	Fungi unclas.	Fungi unclas.	Fungi unclas.
**Otu0151**	-22,879	3,384	-6,761	1.4e-11	3.5e-09	Basidiomycota	Basidiomycota unclas.	Basidiomycota unclas.	Basidiomycota unclas.	Basidiomycota unclas.	Basidiomycota unclas.
**Otu0128**	-23,447	3,384	-7,028	2.1e-12	6.4e-10	Zygomycota	Zygomycota class Incertae sedis	Mortierellales	Mortierellaceae	Mortierella	Mortierella ambigua
**Otu0056**	-24,053	3,383	-7,598	3.0e-14	1.2e-11	Basidiomycota	Agaricomycetes	Cantharellales	Ceratobasidiaceae	Ceratobasidium	Ceratobasidium sp
