# Supplementary material for: Changes in the Microbial Composition of the Rhizosphere of Hop Plants Affected by Verticillium Wilt Caused by Verticillium nonalfalfae
Source: Microorganisms. 2023 Jul 16;11(7):1819. doi: 10.3390/microorganisms11071819 (PMC10385175; doi:10.3390/microorganisms11071819)
Supplement: Supplementary file 1 [file microorganisms-11-01819-s001.zip › Supplementary Material-Table S8.pdf]

Supplementary Material. Table S8.- Fungal OTUs with significant differencial abundances between healthy (HR) and diseased rizhosphere (DR) soils. OTUs with a positive LFC are highlighted on a yellow background while OTUs with a negative LFC are highlighted on a blue background. LFC: log2-fold changes; SE: standard error; *p*-values were adjusted with the False Discovery Rate (FDR) method; unclas.: unclassified

OTU	LFC	LFC (SE)	Wald statistic	*p*-value	FDR	Phylum	Class	Order	Family	Genus	Species
**Otu0092**	24,311	3,384	7,185	6.7e-13	6.4e-10	Basidiomycota	Agaricomycetes	Auriculariales	unclas. Auriculariales	unclas. Auriculariales	Auriculariales sp
**Otu0163**	23,237	3,384	6,867	6.6e-12	2.5e-09	Basidiomycota	Agaricomycetes	Agaricales	Marasmiaceae	Calyptella	Calyptella sp.
**Otu0116**	22,987	3,384	6,793	1.1e-11	3.0e-09	Basidiomycota	Agaricomycetes	Cantharellales	Ceratobasidiaceae	Thanatephorus	Thanatephorus cucumeris
**Otu0283**	21,788	3,385	6,436	1.2e-10	2.3e-08	Basidiomycota	Agaricomycetes	Agaricomycetes unclas.	Agaricomycetes unclas.	Agaricomycetes unclas.	Agaricomycetes unclas.
**Otu0339**	21,216	3,386	6,266	3.7e-10	5.0e-08	Fungi unclas.	Fungi unclas.	Fungi unclas.	Fungi unclas.	Fungi unclas.	Fungi unclas.
**Otu0460**	20,34	3,388	6,003	1.9e-09	2.0e-07	Ascomycota	Eurotiomycetes		Trichocomaceae	Paecilomyces	Paecilomyces penicillatus
**Otu0161**	8,42	1,578	5,336	9.5e-08	9.0e-06	Glomeromycota	Glomeromycetes	Glomerales	Glomeraceae	Funneliformis	Funneliformis mosseae
**Otu0211**	7,961	1,625	4,897	9.7e-07	8.8e-05	Ascomycota	Sordariomycetes	Sordariales	Lasiosphaeriaceae	Lasiosphaeris	Lasiosphaeris hispida
**Otu0321**	7,49	1,758	4,26	2.0e-05	1.6e-03	Chytridiomycota	Chytridiomycetes	Spizellomycetales	Spizellomycetaceae	Powellomyces	Powellomyces sp
**Otu0330**	5,68	1,675	3,391	7.0e-04	3.8e-02	Chytridiomycota	Chytridiomycetes	Spizellomycetales	Spizellomycetaceae	Spizellomyces	Spizellomyces dolichospermus
**Otu0075**	4,637	1,031	4,497	6.9e-06	5.9e-04	Ascomycota	Ascomycota unclas.	Ascomycota unclas.	Ascomycota unclas.	Ascomycota unclas.	Ascomycota unclas.
**Otu0113**	4,262	0,998	4,27	2.0e-05	1.5e-03	Fungi unclas.	Fungi unclas.	Fungi unclas.	Fungi unclas.	Fungi unclas.	Fungi unclas.
**Otu0096**	3,257	0,726	4,488	7.2e-06	5.9e-04	Fungi unclas.	Fungi unclas.	Fungi unclas.	Fungi unclas.	Fungi unclas.	Fungi unclas.
**Otu0125**	3,192	0,938	3,405	6.6e-04	3.7e-02	Ascomycota	Leotiomycetes	Leotiomycetes unclas.	Leotiomycetes unclas.	Leotiomycetes unclas.	Leotiomycetes unclas.
**Otu0037**	3,006	0,821	3,66	2.5e-04	1.7e-02	Fungi unclas.	Fungi unclas.	Fungi unclas.	Fungi unclas.	Fungi unclas.	Fungi unclas.
**Otu0021**	-1,902	0,576	-3,3	9.7e-04	5.0e-02	Ascomycota	Sordariomycetes	Sordariales	Lasiosphaeriaceae	Zopfiella	Zopfiella unclas.
**Otu0121**	-2,639	0,743	-3,552	3.8e-04	2.3e-02	Ascomycota	Sordariomycetes	Sordariales	Chaetomiaceae	Humicola	Humicola grisea var. grisea
**Otu0205**	-4,718	1,202	-3,926	8.6e-05	5.9e-03	Basidiomycota	Tremellomycetes	Trichosporonales	Trichosporonaceae	Trichosporon	Trichosporon laibachii
**Otu0215**	-5,186	1,426	-3,637	2.8e-04	1.7e-02	Zygomycota	Zygomycota class Incertae sedis	Mortierellales	Mortierellaceae	Mortierella	Mortierella unclas.
**Otu0193**	-5,374	1,342	-4,004	6.2e-05	4.4e-03	Ascomycota	Ascomycota unclas.	Ascomycota unclas.	Ascomycota unclas.	Ascomycota unclas.	Ascomycota unclas.
**Otu0099**	-5,54	0,962	-5,759	8.4e-09	8.4e-07	Basidiomycota	Tremellomycetes	Tremellales	Tremellales family Incertae sedis	Cryptococcus	Cryptococcus podzolicus
**Otu0422**	-6,054	1,797	-3,37	7.5e-04	4.0e-02	Ascomycota	Eurotiomycetes	Eurotiomycetes unclas.	Eurotiomycetes unclas.	Eurotiomycetes unclas.	Eurotiomycetes unclas.
**Otu0218**	-6,081	1,721	-3,532	4.1e-04	2.4e-02	Ascomycota	Ascomycota unclas.	Ascomycota unclas.	Ascomycota unclas.	Ascomycota unclas.	Ascomycota unclas.
**Otu0407**	-6,361	1,844	-3,451	5.6e-04	3.2e-02	Chytridiomycota	Chytridiomycetes	Rhizophydiales	Rhizophydiales unclas.	Rhizophydiales unclas.	Rhizophydiales unclas.
**Otu0352**	-7,31	1,75	-4,177	3.0e-05	2.2e-03	Basidiomycota	Tremellomycetes	Trichosporonales	Trichosporonaceae	Trichosporon	Trichosporon unclas.
**Otu0531**	-20,453	3,387	-6,038	1.6e-09	1.7e-07	Chytridiomycota	Chytridiomycetes	Rhizophlyctidales	Rhizophlyctidaceae	Rhizophlyctis	Rhizophlyctis rosea
**Otu0346**	-20,953	3,388	-6,185	6.2e-10	7.4e-08	Basidiomycota	Agaricomycetes	Agaricales	Tricholomataceae	Melanoleuca	Melanoleuca communis
**Otu0468**	-21,026	3,388	-6,207	5.4e-10	6.9e-08	Basidiomycota	Agaricomycetes	Agaricomycetes unclas.	Agaricomycetes unclas.	Agaricomycetes unclas.	Agaricomycetes unclas.

**Otu0453**	-21,263	3,387	-6,278	3.4e-10	5.0e-08	Basidiomycota	Agaricomycetes	Agaricales	Mycenaceae	Mycena	Mycena unclas.
**Otu0493**	-21,28	3,387	-6,283	3.3e-10	5.0e-08	Basidiomycota	Agaricomycetes	Agaricales	Mycenaceae	Mycena	Mycena unclas.
**Otu0308**	-21,623	3,386	-6,386	1.7e-10	2.9e-08	Ascomycota	Ascomycota unclas.	Ascomycota unclas.	Ascomycota unclas.	Ascomycota unclas.	Ascomycota unclas.
**Otu0344**	-21,963	3,385	-6,487	8.7e-11	1.8e-08	Basidiomycota	Agaricomycetes	Agaricales	Mycenaceae	Mycena	Mycena unclas.
**Otu0291**	-22,425	3,385	-6,625	3.5e-11	8.2e-09	Glomeromycota	Glomeromycetes	Glomerales	Glomerales unclas.	Glomerales unclas.	Glomerales unclas.
**Otu0173**	-23,077	3,384	-6,819	9.2e-12	2.9e-09	Chytridiomycota	Chytridiomycetes	Spizellomycetales	Spizellomycetaceae	Spizellomyces	Spizellomyces unclas.
**Otu0151**	-23,336	3,384	-6,896	5.4e-12	2.5e-09	Basidiomycota	Basidiomycota unclas.	Basidiomycota unclas.	Basidiomycota unclas.	Basidiomycota unclas.	Basidiomycota unclas.
**Otu0149**	-23,447	3,384	-6,929	4.3e-12	2.5e-09	Ascomycota	Ascomycota unclas.	Ascomycota unclas.	Ascomycota unclas.	Ascomycota unclas.	Ascomycota unclas.
**Otu0056**	-26,179	3,383	-7,737	1.0e-14	1.9e-11	Basidiomycota	Agaricomycetes	Cantharellales	Ceratobasidiaceae	Ceratobasidium	Ceratobasidium sp
