# Supplementary material for: Changes in the Microbial Composition of the Rhizosphere of Hop Plants Affected by Verticillium Wilt Caused by Verticillium nonalfalfae
Source: Microorganisms. 2023 Jul 16;11(7):1819. doi: 10.3390/microorganisms11071819 (PMC10385175; doi:10.3390/microorganisms11071819)
Supplement: Supplementary file 1 [file microorganisms-11-01819-s001.zip › Supplementary Material-Table S9.pdf]

Supplementary Material. Table S9.- Bacterial OTUs with significant differential abundances between healthy bulk (HB) and healthy rhizosphere (HR) soils. OTUs with a positive LFC are highlighted on a yellow background while OTUs with a negative LFC are highlighted on a blue background. LFC: log2-fold changes; SE: standard error; *p*-values were adjusted with the False Discovery Rate (FDR) method; unclas.: unclassified

OTU	LFC	LFC (SE)	Wald statistic	*p*-value	FDR	Phylum	Kingdom	Class	Order	Family	Genus
**Otu00536**	2,524	0,473	5,332	9,72E-08	3,65E-04	Bacteria	Bacteria_unclas.	Bacteria_unclas.	Bacteria_unclas.	Bacteria_unclas.	Bacteria_unclas.
**Otu00534**	2,377	0,464	5,125	2,97E-07	8,93E-04	Bacteria	Verrucomicrobiota	Verrucomicrobiae	Opitutales	Opitutaceae	Opitutaceae_unclas.
**Otu00283**	1,262	0,298	4,235	2,28E-05	3,82E-02	Bacteria	Myxococcota	Polyangia	Polyangiales	Polyangiaceae	Polyangiaceae_unclas.
**Otu00166**	-1,709	0,376	-4,542	5,57E-06	1,40E-02	Bacteria	Proteobacteria	Gammaproteobacteria	Burkholderiales	Alcaligenaceae	Alcaligenaceae_ge
**Otu00010**	-1,900	0,290	-6,543	6,05E-11	4,55E-07	Bacteria	Actinobacteriota	Actinobacteria	Micrococcales	Micrococcaceae	Pseudarthrobacter
**Otu00312**	-2,515	0,576	-4,367	1,26E-05	2,36E-02	Bacteria	Patescibacteria	Saccharimonadia	Saccharimonadales	Saccharimonadales_fa	Saccharimonadales_ge
**Otu00669**	-3,000	0,679	-4,416	1,00E-05	2,16E-02	Bacteria	Actinobacteriota	Actinobacteria	Glycomycetales	Glycomycetaceae	Glycomyces
**Otu00552**	-3,273	0,520	-6,300	2,98E-10	1,49E-06	Bacteria	Verrucomicrobiota	Verrucomicrobiae	Verrucomicrobiales	Rubritaleaceae	Luteolibacter
**Otu00444**	-5,112	0,747	-6,840	7,92E-12	1,19E-07	Bacteria	Patescibacteria	Saccharimonadia	Saccharimonadales	Saccharimonadaceae	Saccharimonadaceae_unclas.
